# *Orthogonius* species and diversity in Thailand (Coleoptera, Caraboidea, Orthogoniini), a result from the TIGER project

**DOI:** 10.3897/zookeys.164.1992

**Published:** 2012-01-11

**Authors:** Mingyi Tian, Thierry Deuve, Ron Felix

**Affiliations:** 1 Department of Entomology, College of Natural Resources and Environment, South China Agricultural University, Guangzhou, Guangdong 510640, China; 2 Muséum National d’Histoire Naturelle, Département de Systématique et Evolution, Muséum/CNRS UMR 7205, Entomologie, Case Postale 50, 57 rue Cuvier, F-75231 Paris cedex 05, France; 3 Hazelaarlaan 51, 5056 XP, Berkel Enschot, The Netherlands

**Keywords:** Coleoptera, Caraboidea, Orthogoniini, *Orthognius*, new species, new record, Thailand

## Abstract

The carabid genus *Orthogonius* MacLeay is treated, based mainly on materials collected in Thailand through the TIGER project (the Thailand Inventory Group for Entomological Research). Among 290 specimens, 20 species are identified in total, 10 of them are new species: *Orthogonius taghavianae* **sp. n.** (Nakhon Nayok: Khao Yai National Park), *Orthogonius coomanioides* **sp. n.** (Phetchabun: Thung Salaeng Luang National Park), *Orthogonius similaris* **sp. n.** (Phetchabun: Thung Salaeng Luang National Park; Loei: Phu Kradueng National Park), *Orthogonius setosopalpiger* **sp. n.** (Phetchabun: Thung Salaeng Luang National Park), *Orthogonius gracililamella* **sp. n.** (Loei: Phu Kradueng National Park; Chaiyaphum: Tat Tone National Park), *Orthogonius pseudochaudoiri* **sp. n.** (Phetchabum: Thung Salaeng Luang National Park; Nakhon Nayok: Khao Yai National Park), *Orthogonius constrictus* **sp. n.** (Phetchabum: Thung Salaeng Luang National Park), *Orthogonius pinophilus* **sp. n.** (Phetchabum: Thung Salaeng Luang National Park), *Orthogonius vari* **sp. n.** (Cambodia: Siem Reap; Thailand: Ubon Ratchathani: Pha Taem National Park; Phetchabun: Thung Salaeng Luang National Park) and *Orthogonius variabilis* **sp. n.** (Thailand: Phetchabun: Thung Salaeng Luang National Park; Nakhon Nayok: Khao Yai National Park; Phetchabun: Nam Nao National Park; China: Yunnan). In addition, *Orthogonius mouhoti* Chaudoir, 1871 and *Orthogonius kirirom* Tian & Deuve, 2008 are recorded in Thailand for the first time. In total, 30 species of *Orthogonius* have been recorded from Thailand, indicating that Thailand holds one of the richest *Orthogonius* faunas in the world. A provisional key to all Thai species is provided. A majority of Thai *Orthogonius* species are endemic. Among the ten national parks in which orthogonine beetles were collected, Thung Salaeng Luang holds the richest fauna, including 16 species.

## Introduction

Thailand has a diversity of habitat types, including various kind of forests (tropical rain, dry or semi-evergreen, montane evergreen, coniferous, swamp, including mangroves, and deciduous forests) and savanna. Thailand is a meeting place of many faunal elements including the Himalayan, east Palearctic and Oriental Regions. Faunistically, the country falls within two of the top eight biodiversity hotspots as identified by Myers et al. (2000): Indo-Burma (the majority of the country) and Sundaland (in the southern peninsula).

Since 2006, the TIGER project (the Thailand Inventory Group for Entomological Research) has been organized by Drs Michael Sharkey and Brian Broun (the University of Kentucky, Lexington, USA), by means of collaboration with the Queen Sirikit Botanic Garden in Chang Mai, Thailand. Covering 25 national parks in different regions of Thailand, the project has spanned three years and produced diverse materials available for biodiversity inventory, including 290 specimens of the termitophilous ground beetle genus *Orthogonius*.

Despite the fact that taxonomic research on the tribe Orthogoniini of the ground beetles in the Oriental Region has been carried out continuously since 2000 (Tian and Deuve 2000, 2001, 2003a-c, 2004, 2006a-c, 2007a-b, 2008, 2009, 2010; Abhitha et al. 2009), specimens from the TIGER project represent a surprisingly and unknown diversity of species within Thailand. Among the total of 20 identified species of *Orthogonius*, 10 are new to science and are described and illustrated in the present paper. In addition, *Orthogonius mouhoti* Chaudoir, 1871 and *Orthogonius kirirom* Tian & Deuve, 2008 are newly recorded in Thailand.

## Materials and methods

**Figure 1.**
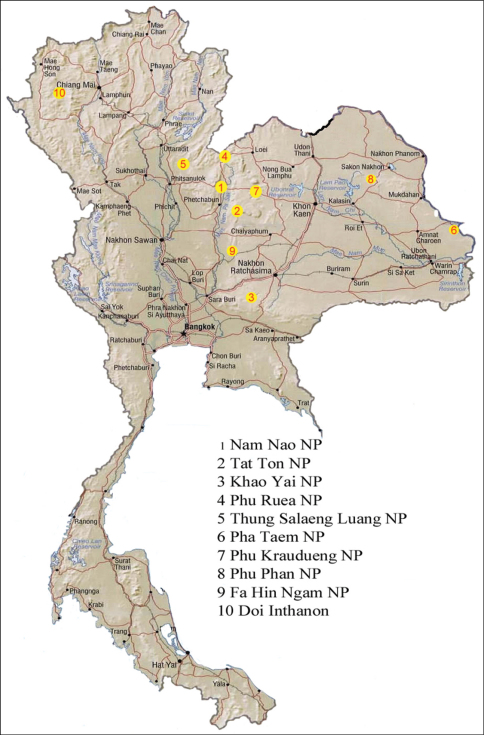
Distribution map of the national parks.

The TIGER project has been carried out in 25 national parks in Thailand over a three year period. A total of 290 specimens of *Orthogonius* used for this study were collected in ten of the parks (Figure 1). Almost all specimens were caught by means of Malaise traps, except a few specimens caught in pan traps or extracted from litter samples. In addition, 98 specimens of *Orthogonius variabilis* sp. n. were collected from Bannahe Nature Reserve, southern Yunnan, China. Other *Orthogonius* specimens were borrowed from the Muséum National d’Histoire Naturelle, Paris (MNHN), from the Institut royal des Sciences naturelles de Belgique, Brussels (IRSNB), from Naturhistorisches Museum, Basel (NHMB), and the Museum of Natural History, London (MNHL) for comparative study.

All specimens were dry mounted. Dissections, drawings, and observations were made using a binocular Leica MZ75 dissecting microscope. Dissected genital pieces, including the median lobe and parameres of the aedeagus, were glued on small paper cards and then pinned under the specimen from which they were removed. Digital pictures were originally taken with Canon EOS 40D camera, and then treated by means of CombineZP and Photoshop softwares.

Abbreviations for measurements were the same as in Tian and Deuve (2006a). The specimen depository is as follow:

**CRF** Collection Ron Felix, Berkel Enschot (the Netherlnads)

**IOZ** Institute of Zoology, Chinese Academy of Sciences, Beijing (China)

**IRSNB** Institut royal des Sciences naturelles de Belgique, Brussels (Belgium)

**MNHN** Muséum National d’Histoire Naturelle, Paris (France)

**QSBG** the Queen Sirikit Botanic Garden, Chang Mai (Thailand)

**SCAU** South China Agricultural University, Guangzhou (China)

## Taxonomic treatment

### Orthogonius taghavianae

Tian, Deuve & Felix sp. n.

urn:lsid:zoobank.org:act:2C0C2E6E-EBC1-4888-AB48-52063D459855

http://species-id.net/wiki/Orthogonius_taghavianae

Figures 2 16

#### Diagnosis.

Large sized, even elytra intervals much wider than odd ones, and covered with dense and coarse punctures, head and pronotum intricately wrinkled or striate; aedeagus distinctly constricted at subapex in dorsal view.

**Figures 2–5.**
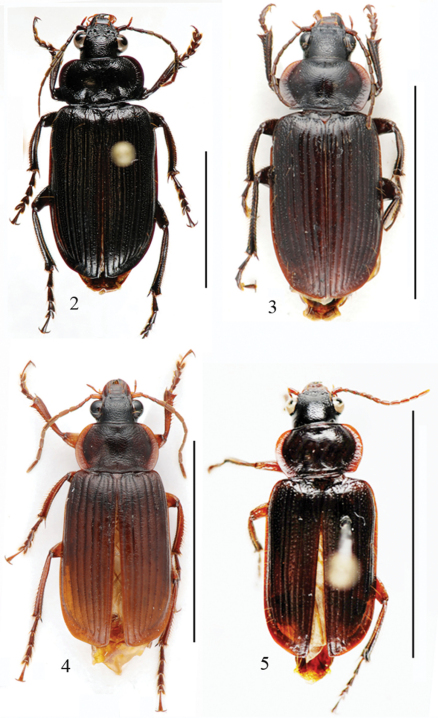
Habitus of *Orthogonius* spp. n. **2** *Orthogonius taghavianae* sp. n. holotype **3** *Orthogonius coomanioides* sp. n. holotype **4** *Orthogonius similaris* sp. n. paratype **5** *Orthogonius setosopalpiger* sp. n. holotype. Scale bar: 10 mm.

Length: 19.0 mm; width: 7.6 mm. Habitus as in Figure 2.

#### Description.

Head and pronotum densely and intricately wrinkled, impunctate, elytra with even intervals densely punctate; microsculptural meshes densely isodiametric on elytra, and irregular on pronotum.

Head moderate, slightly longer than wide (HW/HL=1.05), eyes large, strongly prominent, frons rather flat, vertex convex, neck well-marked; labrum straight at frontal margin, sexsetose, clypeus more or less square, bisetose; palpi normal; maxillar palpomeres 3 and 4 subequal in length, labial palpomere 2 longer than palpomere 3; ligula narrow, bisetose at apex; mentum and submentum each with a pair of setae; palpiger short, asetose; antennae extending to 1/7 of elytra from base, densely pubescent from basal 1/4 of antennomere 4; antennomere 3 as long as 4.

Pronotum strongly transverse, PW/PL=1.85, widest at about middle; both fore and hind angles broadly rounded; front and hind margins well beaded; lateral expanded margins wide, smooth and reflexed; transverse impressions well marked, basal foveae moderate.

Elytra broad and strongly convex; EL/EW=1.58; sides nearly parallel; widest at about middle, apex roundly truncate, strongly sinuate near inner angle which is pointed; base well bordered; shoulders more or less square; striae deep, punctate-striate, intervals convex; even intervals much wider than odd ones (almost twice) and with coarser punctures which extended to the subapical portion, odd intervals with a row of fine and sparse punctures; interval 3 with three discal setiferous pores, and additional two at apical portion; interval 5 with two setae near base; interval 7 narrow and carinate before middle, with seven setiferous pores.

Legs stout, fore tibia with outer angle very sharp and strongly protruded, outer margin distinctly serrate; middle tibia distinctly dilated, and strongly curved in median portion; hind tibia elongate, with tibial spurs short and more or less blunt; hind tarsomere 1 longer than 2, tarsomeres 3 and 4 subequal, tarsomere 4 bilobed; hind femur moderately dilated, with five posterior setae; all tarsal claws pectinate.

Prosternal process well bordered at apex, middle coxa with several setae in median portion; abdominal ventrite VII of male complete at apex.

Male genitalia (Figure 16): Elongate, enlarged at about middle portion, ventral margin sinuate, dorsal opening large and long, abruptly truncate near apex; in dorsal view, apical part narrow, distinctly constricted before apical lamella, apical lamella elongate, about 2.2 times as long as wide, blunt at apex.

**Female.** Unknown.

#### Remarks.

This species is a member of the *Orthogonius alternans* species group, but with distinctive aedeagal structure.

#### Material examined.

Holotype: male, “Thailand: Nakhon Nayok: Khao Yai NP, entrance of Hnong Pak Chee Trail, 14°27.167'N, 101°21.850'E, 758 m, 5–12.v.2007, Malaise traps, Pong Sandao leg., T2263”, deposited in QSBG.

#### Etymology.

 This new species is named in honour of Ms Azadeh Taghavian, a curator of the Coleoptera collection in MNHN, Paris, in thanks for her help in so many ways.

#### Distribution.

 Thailand. Known only from the type locality.

### Orthogonius coomanioides

Tian, Deuve & Felix sp. n.

urn:lsid:zoobank.org:act:BF1DD07D-514B-4300-B2A6-B088AE086F18

http://species-id.net/wiki/Orthogonius_coomanioides

Figures 3 17

#### Diagnosis.

 Medium sized, even elytra intervals wider than odd intervals, but less than twice as wide; similar to *Orthogonius coomani* Tian & Deuve, 2006, but a little larger, darker, and broader than the latter; in addition, head and eyes rather smaller and less prominent; middle tibiae strongly curved (not distinctly curved in *Orthogonius coomani*); aedeagus stouter, and apical lamella distinctly broader than that of *Orthogonius coomani*.

Length: 13.0-14.0 mm; width: 6.5-6.7 mm. Habitus as in Figure 3.

#### Description.

Head and pronotum densely and intricately wrinkled, impunctate, microsculptural meshes densely isodiametric on head, pronotum and elytra.

Head moderate, slightly longer than wide, eyes rather small, less prominent, labrum distinctly emarginate at frontal margin, sexsetose, clypeus bisetose; palpi normal; mentum and submentum each with a pair of setae; palpiger asetose; antennae extended to the shoulders of elytra, densely pubescent from antennomere 4.

Pronotum strongly transverse, PW/PL=1.70-1.72, widest a little before middle; both fore and hind angles broadly rounded; lateral expanded margins wide, striate and more or less reflexed; transverse impressions well marked, median line clear.

Elytra broad, strongly convex; EL/EW=1.63-1.64; sides parallel; apex roundly truncate; even intervals well bordered at base; striae deep, punctate-striate; intervals convex, even intervals much wider than odd ones (but less than twice as wide) and with coarser punctures extended to apical 1/3 of elytra; interval 3 with only basal and apical setiferous pores, middle pore absent; interval 5 with two setae near base; interval 7 narrow, distinctly carinate, with eight to nine setiferous pores.

Legs moderate, fore tibia with outer angle very sharp and strongly protruded, outer margin hardly serrate; middle tibia distinctly dilated at apex, and strongly curved in median portion; hind tibia elongate, with tibial spurs moderately long, sword-like, sharp; hind femur moderately dilated, with four posterior setae; hind tarsomere 3 (1.2 times) longer than 4, tarsomere 4 deeply emarginate (a little more than half of the joint); all tarsal claws weakly pectinate.

Prosternal process bordered at apex, middle coxa with several setae; ventrite VII of male complete at apex.

Male genitalia (Figure 17): Short, and stout, ventral margin expanded strongly at middle portion, apex distinctly bent ventrally; dorsal opening very wide and long; apical lamella broad, but much longer than wide.

**Female.** Unknown.

#### Remarks.

 This species is closely allied to *Orthogonius coomani*, with differences as mentioned above.

#### Material examined.

Holotype: male, “Thailand: Phetchabun: Thung Salaeng Luang NP, pine forest, 16°35.789'N, 100°52.769'E, 732 m, Malaise trap,15–22.vi.2007, Pongpitak Pranee & Sathit leg., T2059”, in QSBG.

Paratypes: 4 males, “Thailand: Phetchabun: Thung Salaeng Luang NP, pine forest, 16°35.789'N, 100°52.769'E, 732 m, Malaise trap,15–22.vi.2007, Pongpitak Pranee & Sathit leg., T2059”; 4 males, “Thailand: Phetchabum, Thung Salaeng Luang NP, Gang Wang Nam Yen, pine forest, 16°35.789N, 100°52.769E, 723 m, Malaise trap, 6–13.vii.2007, Pongpitak Pranee & Sathit leg., T2068”; 1 male, “Thailand: Phetchabun: Thung Salaeng Luang NP, pine forest; Gang Wang Nam Yen, 16°36.284'N, 100°53.128'E, 749 m, 22–23.vi.2007, Pan traps, Pongpitak & Sathit leg., T2058”; 4 males, “Thailand: Phetchabum, Thung Salaeng Luang NP, Gang Wang Nam Yen, 16°36.284N, 100°53.128E, 749 m, Malaise trap, 29.vi–6.vii.2007, Pongpitak Pranee & Sathit leg. T2069”, in QSBG, MNHN, SCAU and CRF, respectively.

#### Etymology.

 The name refers to the similarity of the new species with *Orthogonius coomani*, which occurring in Vietnam.

#### Distribution.

 Thailand. Known only from the type locality and other nearby site in Thung Salaeng Luang NP.

### Orthogonius similaris

Tian, Deuve & Felix sp. n.

urn:lsid:zoobank.org:act:FD099C32-02ED-487B-949E-7D012B2D212B

http://species-id.net/wiki/Orthogonius_similaris

Figures 4 18

#### Diagnosis.

 A peculiar species with following aspects: medium sized; densely punctate on whole surface; ligula small, but quadrisetose at apex; shape of abdominal ventrite VII in male very similar to that of *Hexachaetus taylorae* Tian & Deuve, 2006.

Length: 14.0 mm; width: 6.0 mm. Habitus as in Figure 4.

#### Description.

 Dark brown or black, but antennae, expanded pronotal margins, palpi, legs and underside surface reddish brown.

Upper surface densely punctate, pronotum with transverse striae, elytra with dense and very short, transverse and granular wrinkles (esp. near base); underside surface smooth and glabrous, polished.

Microsculptural meshes densely isodiametric.

Head stout, as long as wide, eyes very large, and strongly prominent; frons and vertex strongly convex, frontal impressions small and fovea-like, clypeus bisetose, surface even, labrum sexsetose, sides rounded, middle portion slightly emarginate; ligula small and narrow, quadrisetose at apex; palpi slender, subcylindrical, maxillary palpomere 4 longer than 3, palpomere 3 glabrous, except several setae at apex; maxillary palpomere 4 with very short setae; labial palpomere 3 as long as palpomere 2, palpomere 3 with a few setae at base; labial palpomere 2 bisetose in inner margin, and with two or three additional setae at subapex and apex; palpiger asetose, mentum and submentum each with one pair of setae; mentum without median tooth. Antennae moderate, extended to basal 1/4 of elytra; pubescent from basal 1/3 of antennomere 4; antennomere 3 slightly shorter than antennomere 4.

Pronotum transverse, widest at about basal 1/3, PW/PL=1.74, disc slightly and evenly convex, both fore and hind angles broadly rounded, both basal and fore margins beaded, lateral expanded margins rather wide, even and hardly reflexed; fore and hind transverse impressions faint, basal foveae not well marked.

Elytra elongate ovate, EL/EW=1.64; moderately convex, basal border complete, shoulders broadly square; sides more or less parallel at middle, widest at about middle; striae deep, punctate-striate, intervals distinctly convex; intervals subequal in width in middle; apex quite broadly truncate, inner angle nearly rectangular; interval 3 without setiferous pore, interval 7 simple, without pore.

Prosternal process well bordered at apex. Middle and hind coxae smooth and glabrous. Apical margin of abdominal ventrite VII of male deeply and widely emarginate at apical margin, then strongly sinuate at sides behind paramedial setae.

Legs stout. Fore tibia with apical outer angle nearly rectangular, not protruded or pointed; outer margin distinctly serrate; middle tibia evenly curved, gradually dilated towards apex, in lateral view, while slender in dorsal view; hind tibia slender, apical spurs moderate long, sword-like, tarsomere 1 as long as 2, tarsomere 3 longer than 4, tarsomere 4 bilobed; fore tarsi much wider than middle and hind tarsi (which are slender); all tarsal claws strongly pectinate.

Male genitalia (Figure 18): Median lobe of aedeagus quite stout, less expanded at middle portion; apex broadly blunt; dorsal opening wide and long; in dorsal view apical lamella small and sharp.

**Female.** Unknown.

#### Remarks.

 This new species is peculiar in its surface extraordinarily densely punctate, the shape of abdominal ventrite VII, and aedeagal structure. It is similar to *Hexachaetus taylorae* Tian & Deuve, 2006, but differs with the latter by: (1) ligula narrow, quadrisetose (wide and sexsetose in *Hexachaetus taylorae*); (2) pronotum and elytra with dense punctures (sparsely punctate in *Hexachaetus taylorae*); and (3) the apical lamella of aedeagus shorter and broader (longer and narrower in *Hexachaetus taylorae*).

#### Material examined.

Holotype: male, “Thailand: Phetchabun: Thung Salaeng Luang NP: Gang Wang Nam Yen, 750 m, 16°36.587'N, 100°53.395'E; 17–24.v. 2007, Pongpitak Pranee & Sathit leg. T2080”, in QSBG

Paratypes: 1 male, “Thailand: Loei: Phu Kradueng NP, mixed deciduous forest, south of Na Noy Forest Unit, 16°49.099'N, 101°47.624'E, 275 m, 14.xi.2006–18.xi.2006, Litter sample, Suthin Gong-Lasae leg., T1064”; 1 male, “Thailand: Phetchabun: Thung Salaeng Luang NP, Gang Wang Nam Yen, 16°36.587'N, 100°53.395'E, 753 m, 24–31.v.2007, Malaise trap, Pongpitak Pranee & Sathit leg., T2083”; 1 male, “Thailand: Phetchabun: Thung Salaeng Luang NP, Gang Wang Nam Yen, 16°37.531'N, 100°53.745'E, 721 m, 7–14.vi.2007, Malaise trap, Pongpitak Pranee & Sathit leg., T2091”; 2 males, “Thailand: Phitsanulok: Thung Salaeng Luang NP, moist evergreen, 16°50.641'N, 100°52.894'E, 557 m, 11.viii.2006–18.viii.2006, Malaise trap, Pongpitak Pranee leg., T566”; 1 male, “Thailand: Phetchabum, Thung Salaeng Luang NP, Gang Wang Nam Yen, 16°37.178N, 100°.53.504E, 706 m, Malaise trap, 17–24.v.2007, Pongpitak Pranee & Sathit leg. T2081”; 1 male, “Thailand: Phetchabun: Thung Salaeng Luang NP, pine forest; Gang Wang Nam Yen, 16°36.284'N, 100°53.128'E, 749 m, 20–21.vi.2007, Pan traps, Pongpitak & Sathit leg., T2056”; 1 male, “Thailand: Phetchabun: Thung Salaeng Luang NP, pine forest, 16°35.789'N, 100°52.769'E, 732 m, Malaise trap,15–22.vi.2007, Pongpitak Pranee & Sathit leg., T2059”, in QSBG, MNHN, SCAU and CRF, respectively.

#### Etymology.

 The name of this new species refers to its similarity to *Hexachaetus taylorae*.

#### Distribution.

 Thailand. Known only from the type locality and other nearby sites in Thung Salaeng Luang NP.

### Orthogonius setosopalpiger

Tian, Deuve & Felix sp. n.

urn:lsid:zoobank.org:act:E2CE2CC1-4B62-4F0C-976C-ACD79C64CFFA

http://species-id.net/wiki/Orthogonius_setosopalpiger

Figures 5 19

#### Diagnosis.

 Small sized, even elytral intervals densely punctuate; ligula small, bisetose at apex, palpiger with a long seta near base; allied to *Orthogonius grootaerti* Tian & Deuve, 2006 and *Orthogonius angkor* Tian & Deuve, 2006, but smaller.

Length: 11.0 mm; width: 4.5 mm. Habitus as in Figure 5.

#### Description.

Dark brown, antennae, palps, lateral expanded margins of pronotum, underside surface except head reddish brown.

Head and pronotum irregularly wrinkled, impunctate, elytra with even intervals densely punctate, odd ones smooth; microsculptural meshes densely isodiametric on elytra, irregular on head and pronotum.

Head moderate, as long as wide, eyes moderate, strongly prominent, frons rather flat, vertex convex, neck well-marked; labrum deeply emarginate at frontal margin, sexsetose, clypeus more or less square, bisetose, base processed in middle; palpi normal; maxillar palpomeres 3 and 4 subequal, labial palpomere 2 slightly longer than palpomere 3; ligula narrow, bisetose at apex; mentum and submentum each with a pair of setae; palpiger short, with a long seta at base; antennae extended to base of elytra, densely pubescent from basal 1/3 of antennomere 4; antennomere 3 as long as antennomere 4.

Pronotum moderately transverse, PW/PL=1.52, widest at about middle; both fore and hind angles broadly rounded; front and hind margins well beaded; lateral expanded margins wide, almost evenly wide throughout, and slightly reflexed; transverse impressions well marked at base, faint at subapex; basal foveae small.

Elytra broad and strongly convex; EL/EW=1.67; sides nearly parallel; widest at about middle, apex roundly truncate, not sinuate before inner angles; base well bordered; shoulders more or less square; striae deep, punctate-striate, intervals convex; even intervals much wider than odd intervals (almost twice as wide, except interval 4, which is less twice as wide as 3) and with coarser punctures extended to apical 1/4 of elytra, odd intervals with a few fine punctures more or less arranged in a row; interval 3 with three setiferous pores; interval 5 with one seta near base; interval 7 narrow but not carinate throughout, with eleven setiferous pores.

Legs stout, fore tibia with outer angle very sharp and strongly protruded, outer margin slightly subserrate; middle tibia not distinctly curved in median portion, moderately dilated; hind tibia elongate, with apical tibial spurs short and sword-like; tarsomere 1 longer than tarsomere 2, tarsomere 3 slightly longer than 4, tarsomere 4 asymmetrically bilobed; hind femur moderately dilated, with four posterior setae. All tarsal claws strongly pectinate.

Prosternal process well bordered at apex, middle coxa with three or four setae; ventrite VII of male complete at apex.

Male genitalia (Figure 19): Elongate, more or less straight, less sinuate ventrally as in other species, hardly bent towards apex; in dorsal view, apical lamella broad at apex, symmetrical, longer than wide.

**Female.** Unknown.

#### Remarks.

 This species is a member of the *Orthogonius grootaerti* species group. It differs from *Orthogonius grootaerti* and *Orthogonius angkor* by its: (1) smaller sized; (2) aedeagus more elongate and apical lamella longer than in both above species; and (3) hind femur 4-setose posteriorly (6-setose in *Orthogonius grootaerti* and *Orthogonius angkor*).

#### Type material.

Holotype: male, “Thailand: Phetchabun: Thung Salaeng Luang NP: Gang Wang Nam Yen, 750 m, 16°37.178'N, 100°5.504'E Pan traps, 23–24.v. 2007, Pongpitak Pranee & Sathit leg., T2079”, in QSBG.

#### Etymology.

 The name of this new species refers to its setose palpiger.

#### Distribution.

 Thailand. Known only from the type locality.

### Orthogonius pangi

Tian & Deuve, 2006

http://species-id.net/wiki/Orthogonius_pangi

#### Material examined.

 1 male, “21?6”, Thailand: detailed data unclear because of damaged label; either from Khao Yai National Park if the label is “2126”, or from Pha Taem National Park if is “2186”; 1 male, “Thailand: Nakhon Nayok: Khao Yai NP, Lum Ta Kong View Point, 14°25.762'N, 101°23.527'E, 732 m, 5–12.iv.2007, Malaise trap, Wirat Sukho leg., T2122”; 1 male, “Thailand: Nakhon Nayok: Khao Yai NP, Lum Ta Kong View Point, 14°25.82'N, 101°23.754'E, 744 m, 26.iv.2007–2.v.2007, Malaise trap, Pong Sandao leg., T2132”, in QSBG and MNHN respectively.

#### Distribution.

 Thailand.

### Orthogonius huananoides

Tian & Deuve, 2006

http://species-id.net/wiki/Orthogonius_huananoides

#### Material examined.

 6 males, “Thailand: Nakhon Nayok: Khao Yai NP, Lum Ta Kong View Point, 14°25.82'N, 101°23.754'E, 744 m, 19–26.iv.2007, Malaise trap, Wirat Sukho leg., T2129”; 1 male, “Thailand: Nakhon Nayok: Khao Yai NP, Lum Ta Kong View Point, 14°25.565'N, 101°23.442'E, 726 m, 19–26.iv.2007, Malaise trap, Wirat Sukho leg., T2127”; 8 males, “21?6”, Thailand: detail data unclear because of label damaged, either from Khao Yai National Park if the label is “2126”, or from Pha Taem National Park if is “2186”; 2 males, “Thailand: Nakhon Nayok: Khao Yai NP, Lum Ta Kong View Point, 14°25.565'N, 101°23.442'E, 726 m, 26.iv.2007–2.v.2007, Malaise trap, Pong Sandao leg., T2130”; 8 males, “Thailand: Nakhon Nayok: Khao Yai NP, Lum Ta Kong View Point, 14°25.762'N, 101°23.527'E, 732 m, 26.iv.2007–2.v.2007, Malaise trap, Wirat Sukho leg., T2131”; 2 males, “Thailand: Nakhon Nayok: Khao Yai NP, entrance of Hnong Pak Chee Trail, 14°27.115'N, 101°21.951'E, 733 m, 5–12.v.2007, Malaise traps, Wirat Sukho leg., T2264”; 5 males, “Thailand: Nakhon Nayok: Khao Yai NP, entrance of Hnong Pak Chee Trail, 14°27.167'N, 101°21.85'E, 758 m, 5–12.v.2007, Malaise traps, Pong Sandao leg., T2263”; 1 male, “Thailand: Nakhon Nayok: Khao Yai NP, entrance of Hnong Pak Chee Trail, 14°27.115'N, 101°21.951'E, 733 m, 19–26.v.2007, Malaise traps, Pong Sandao leg., T2270”; 1 male, “Thailand: Nakhon Nayok: Khao Yai NP, Lum Ta Kong View Point, 14°25.820'N, 101°23.754'E, 744 m, 5–12.iv.2007, Malaise trap, Pong Sandao, leg., T2123”; 6 males, “Thailand: Nakhon Nayok: Khao Yai NP, Lum Ta Kong View Point, 14°25.82'N, 101°23.754'E, 744 m, 26.iv.2007–2.v.2007, Malaise trap, Pong Sandao leg., T2132”; 1 male, “Thailand: Nakhon Nayok: Khao Yai NP, entrance of Hnong Pak Chee Trail, 14°27.115'N, 101°21.951'E, 733 m, 10–11.v.2007, Pan traps, Wirat Sukho leg., T2261”; 2 males, “Thailand: Nakhon Nayok: Khao Yai NP, entrance of Hnong Pak Chee Trail, 14°27.119'N, 101°21.482'E, 699 m, 12–19.v.2007, Malaise traps, Wirat Sukho leg., T2268”; 3 males, “Thailand: Nakhon Nayok: Khao Yai NP, Lum Ta Kong View Point, 14°25.762'N, 101°23.527'E, 732 m, 5–12.iv.2007, Malaise trap, Wirat Sukho leg., T2122”; 2 males, “Thailand: Nakhon Nayok: Khao Yai NP, entrance of Hnong Pak Chee Trail, 14°27.167'N, 101°21.85'E, 758 m, 19–26.v.2007, Malaise traps, Wirat Sukho leg., T2269”; 1 male, “Thailand: Nakhon Nayok: Khao Yai NP, entrance of Hnong Pak Chee Trail, 14°27.115'N, 101°21.951'E, 733 m, 12–19.v.2007, Malaise traps, Pong Sandao leg., T2267”; 8 males, “Thailand: Nakhon Nayok: Khao Yai NP, Lum Ta Kong View Point, 14°25.762'N, 101°23.527'E, 732 m, 12–19.iv.2007, Malaise trap, Wirat Sukho leg., T2125”; 1 male, “Thailand: Nakhon Ratchasima: Khao Yai NP, Cobra zone near fire protection office, 14°28.524'N, 101°22.928'E, 757 m, 5–12.vi.2007, Malaise trap, Pong Sandao leg., T2221”; 1 male, “Thailand: Nakhon Nayok: Khao Yai NP, entrance of Hnong Pak Chee Trail, 14°27.115'N, 101°21.951'E, 733 m, 11–12.v.2007, Pan traps, Pong Sandao leg., T2262”; 1 male, “Thailand: Nakhon Ratchasima: Khao Yai NP, Cobra zone near fire protection office, 14°28.524'N, 101°22.928'E, 757 m, 5–12.vi.2007, Malaise trap, Pong Sandao leg., T2221”; 1 male, “Thailand: Nakhon Nayok: Khao Yai NP, entrance of Hnong Pak Chee Trail, 14°27.115'N, 101°21.951'E, 733 m, 6–7.v.2007, Pan traps, Wirat Sukho leg., T2257”; 2 males, “Thailand: Nakhon Nayok: Khao Yai NP, Lum Ta Kong View Point, 14°25.565'N, 101°23.442'E, 726 m, 12–19.iv.2007, Malaise trap, Wirat Sukho leg., T2124”; 1 male, “Thailand: Phetchabun: Thung Salaeng Luang NP, pine forest; Gang Wang Nam Yen, 16°36.284'N, 100°53.128'E, 749 m, 22–23.vi.2007, Pan traps, Pongpitak & Sathit leg., T2058”; 1 male, “Thailand: Nakhon Nayok: Khao Yai NP, Lum Ta Kong View Point, 14°25.762'N, 101°23.527'E, 732 m, 19–26.iv.2007, Malaise trap, Pong Sandao leg., T2128”; 1 male, “Thailand: Ubon Ratchathani: Pha Taem NP, west of HuayPok substation, 15°37.212'N, 105°36.903'E, 438 m, 4–11.iv.2007, Malaise trap, Bunlu Sapsiri leg., T2159”; 2 males, “Thailand: Nakhon Nayok: Khao Yai NP, Lum Ta Kong View Point, 14°25.82'N, 101°23.754'E, 744 m, 12–19.iv.2007, Malaise trap, Wirat Sukho leg., T2126”, in QSBG, MNHN, SCAU and CRF, respectively.

#### Distribution.

 Thailand and Vietnam.

### Orthogonius gracililamella

Tian, Deuve & Felix sp. n.

urn:lsid:zoobank.org:act:AB7D1670-CFA3-4273-8EBF-9B489C4D699A

http://species-id.net/wiki/Orthogonius_gracililamella

Figures 6 20

#### Diagnosis.

 Moderate or small sized, member of the *Orthogonius longicornis* species group, eyes very large; mentum asetose; apex of elytra shortly and obliquely truncate at inner margin of the tip to form an obvious sutural angle between elytra; labrum slightly emarginate at frontal margin; prosternal process bordered at apex, base of elytra complete; hind tarsomere 4 slightly emarginate at apex, hind femur quite slender, with two setae posteriorly, all tarsal claws strongly pectinate; ventrite VII very slightly emarginate at apical margin; aedeagus with apical lamella long and parallel-sided.

**Figures 6–9.**
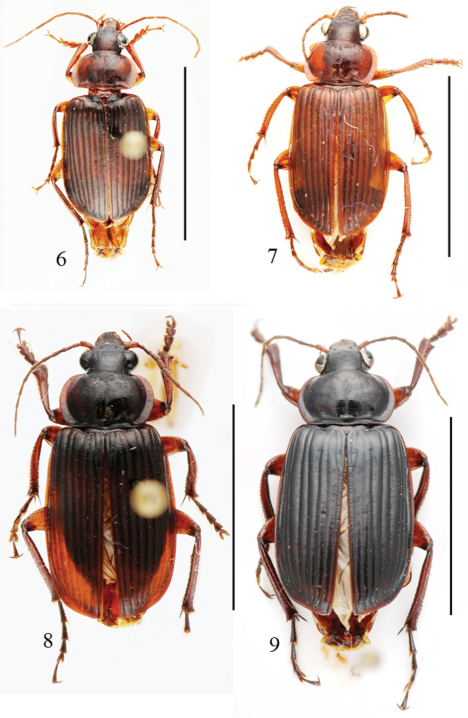
Habitus of *Orthogonius* spp. n. **6** *Orthogonius gracililamella* sp. n. holotype **7–9** *Orthogonius pseudochaudoiri* sp. n. paratypes. Scale bar: 10 mm.

Length: 12.0–13.0 mm; width: 5.0–5.5 mm. Habitus as in Figure 6.

#### Description.

Light dark brown (HT) to black (PT), palps, antennae, lateral expanded margin of pronotum, tibiae, femora and trochanters lighter.

Wrinkles and punctures: surface impunctate except elytral intervals 3, 5 and 7 with tiny, and sparse punctures arranged as a row, head wrinkled, pronotum faintly striate.

Microsculptural meshes isodiametric on elytra, head and pronotum.

Head as long as wide, eyes very large, strongly prominent; frons and vertex convex, frontal impressions faint, clypeus bisetose, even; labrum quadrate, sexsetose, frontal margin slightly emarginate at frontal margin; mandibles well developed; ligula small and narrow, bisetose at apex; palpi slender, subcylindrical, maxillary palpomere 3 as long as 4, glabrous; labial palpomere 2 longer than 3, 2-setose in inner margin; labial palpomere 3 sparsely pubescent; palpiger asetose, mentum asetose, and submentum with one pair of setae; mentum without median tooth. Antennae extended to near basal 1/4 of elytra; pubescent after basal 1/4 of antennomere 4, antennomeres 4–5, slightly dilated, antennomere 3 shorter than 4 (about 0.7 times as its length).

Pronotum strongly transverse, PW/PL=1.56, moderately convex; sides evenly rounded, widest at about middle, both basal and fore margins beaded, lateral expanded margins well defined, uneven, slightly reflexed; fore and hind angles rounded; fore transverse impression unclear, hind one distinct, basal foveae small, but well marked, middle line clear.

Elytra ovate, EL/EW=1.60, convex, basal border complete, sides slightly expanded, not parallel at middle, striae deep, intervals convex, intervals subequal in width in middle; apex roundly truncate, but shortly and obliquely truncate at inner margin of the tip to form an obvious sutural angle between elytra; interval 3 with three setae, all are well marked; interval 7 not carinate, without seta.

Middle coxa glabrous in median portion; hind coxa with two setae. Legs moderate, fore tiba with outer angle rectangular, serrate on outer margins, apical margin oblique; middle tibia straight in middle, abruptly dilated at apex; hind tibiae slender, slightly dilated only at apex; hind tibial spurs very long and sharp; tarsomere 4 much longer than tarsomere 3 (almost 1.25 times as long), tarsomere 4 very shallowly emarginate at apex; hind femora rather slender, with two setae posteriorly; all tarsal claws strongly pectinate.

Prosternal process well bordered at apex. Apical margin of abdominal ventrite VII very shallowly and slightly emarginate.

Male genitalia (Figure 20): Moderately elongate, ventral margin more or less sinuate ventrally, apex pointed in lateral view; in dorsal view, apical lamella very long and nearly parallel-sided.

**Female.** Unknown.

#### Remarks.

 The apical portion of the aedeagus is very elongate, a little more twisted in the holotype than in the paratype, and the apical lamella is slender and parallel-sided, with the apex broadly rounded.

#### Material examined.

Holotype: male, “Thailand: Loei: Phu Kradueng NP, Huay Lao Kao, 16°52.442N, 101°50.706E, 280 m, Malaise trap, 29–30.viii.2006, Sutin Khonglassae leg. T490”, in QSBG.

Paratype: 1 male, “Thailand: Chaiyaphum: Tat Tone NP, water supply station at Taad Fah waterfall, 15°56.468N, 102°05.855E, 245 m, Malaise trap, 5–12.ix.2006, Tawit Jaruphan & Orawan Budsawong leg., T686”, in QSBG.

#### Etymology.

 The name refers to the long and narrow apical lamella of aedeagus.

#### Distribution.

 Thailand. Known only from the type locality.

### Orthogonius pseudochaudoiri

Tian, Deuve & Felix sp. n.

urn:lsid:zoobank.org:act:597B2644-6DB2-48DC-81A7-24BC6E304379

http://species-id.net/wiki/Orthogonius_pseudochaudoiri

Figures 7–9 21

#### Diagnosis.

 Small to medium sized, labrum straight at frontal margin; prosternal process well bordered at apex, abdominal ventrite VII slightly emarginate at apical margin; very similar to *Orthogonius mouhoti* Chaudoir, 1871, but apical lamella of the aedeagus much longer than that of the latter species.

Length: 12.5–16.0 mm; width: 5.5–7.0 mm. Habitus as in Figures 7–9.

#### Description.

Dark brown to black, lateral expanded margin of pronotum, antennae, mouthparts palpi, legs and underside surface reddish brown.

Wrinkles and punctures: surface smooth and impunctate; head and pronotum faintly striate, odd elytral intervals (3, 5, 7) with distinct fine punctures which are irregularly rowed; surface strongly shiny.

Microsculptural meshes densely isodiametric, clear on elytra, but faint on pronotum and head.

Head stout, as long as wide; eyes very large, strongly prominent, frons and vertex moderately convex, frontal impressions small, short, fovea-like, clypeus bisetose, rather even, labrum sexsetose, nearly straight at apical margin; ligula very small and narrow, bisetose at apex; palpi slender, subcylindrical, normal; palpiger asetose, mentum without median tooth, asetose, mentum and submentum each with one pair of setae. Antennae slender, extended beyond basal 1/3 of elytra, pubescent from apical 2/3 of antennomere 4; antennomeres 3, 4 and 5 subequal in length; antennomeres 1–3 glabrous; antennomeres 4–6 distinctly expanded laterally.

Pronotum strongly transverse, PW/PL=1.88–1.90, sides evenly rounded, widest at about middle, both basal and fore margins beaded, lateral expanded margins well defined, wide and even, flat and smooth; both fore and hind angles rounded; disc strongly convex, fore transverse impression faint, basal one moderate, basal foveae small.

Elytra broadly ovate (EL/WL=1.55–1.57), strongly convex, basal border well complete; sides slightly expanded in middle portion, hardly parallel-sided, widest at middle; striae deep, punctate-striate, intervals distinctly convex; intervals 2–5 subequal in width, interval 6 much wider than 5; odd intervals with more distinct fine punctures, irregular in row; apex roundly truncate, inner angle finely toothed, and with a wider sutural angle; interval 3 with three well marked setiferous pores, near striae 3, 2 and 2, respectively; interval 7 simple, wide and not carinate, without seta throughout.

Legs moderate, fore tibia with outer angle nearly rectangular, blunt, outer margin not serrate; middle and hind coxae smooth and glabrous; middle and hind tibia slender, apex slightly dilated, hind apical tibial spurs very long and sharp; tarsomere 3 much longer than 4, tarsomere 4 deeply emarginate at apex (about 1/3 deep as the joint); all tarsal claws strongly pectinate; hind femur with 2 posterior setae on ventral.

Prosternal process well bordered at apex; apical margin of abdominal ventrite VII narrowly and shallowly emarginate in male.

Male genitalia (Figure 21): Very similar to that of *Orthogonius chaudoiri*, straight, and arrowhead-shaped at apex in dorsal view, but more distinctly so than in *Orthogonius chaudoiri*, upper margin less sinuate, and apical lamella in dorsal view much longer and more elongate.

**Female.** Unknown.

#### Remarks.

 This species is very similar to *Orthogonius chaudoiri*, but the apex of its aedeagus is more distinctly arrowhead-shaped than that of *Orthogonius chaudoiri*, less sinuate, and apical lamella much longer; labrum slightly emarginate (straight in latter); body a little more slender; and ventrite VII of male with a small emargination at apical margin.

#### Variability.

Shape of the arrow-headed apex of the aedeagus is variable, wider in some specimens, but narrower in others; however, in all specimens of this species examined, the apical lamella is much longer than that of *Orthogonius chaudoiri*.

#### Material examined.

Holotype: male, “Thailand: Phetchabum, Thung Salaeng Luang NP, Gang Wang Nam Yen, 16°37.531N, 100°53.745E, 721 m, Malaise trap, 17–24.v.2007, Pongpitak Pranee & Sathit leg., T2082”, in QSBG.

Paratypes: 1 male, data as holotype; 6 males, “Thailand: Phetchabun: Thung Salaeng Luang NP, pine forest, 16°35.789'N, 100°52.769'E, 732 m, Malaise trap,15–22.vi.2007, Pongpitak Pranee & Sathit leg., T2059”; 3 males, “Thailand: Phetchabum, Thung Salaeng Luang NP, Gang Wang Nam Yen, 16°36.284N, 100°53.128E, 749 m, Malaise trap, 29.vi–6.vii.2007, Pongpitak Pranee & Sathit leg., T2087”; 2 males, “Thailand: Phetchabun: Thung Salaeng Luang NP, pine forest;G ang Wang Nam Yen, 16°36.284'N, 100°53.128'E, 749 m, 15.vi.2007–18.vi.2007, Litter sample, Pongpitak & Sathit leg., T2050”; 1 male, “Thailand: Phetchabun: Thung Salaeng Luang NP, pine forest; Gang Wang Nam Yen, 16°36.284'N, 100°53.128'E, 749 m, 30.vi.2007–3.vii.2007, Litter sample, Pongpitak & Sathit leg., T2051”; 6 males, “Thailand: Phetchabun: Thung Salaeng Luang NP, pine forest; Gang Wang Nam Yen, 16°36.284'N, 100°53.128'E, 749 m, 16–17.vi.2007, Pan traps, Pongpitak & Sathit leg., T2052”; 1 male, “Thailand: Phetchabun: Thung Salaeng Luang NP, pine forest; Gang Wang Nam Yen, 16°36.284'N, 100°53.128'E, 749 m, 18–19.vi.2007, Pan traps, Pongpitak & Sathit leg., T2054”; 5 males, “Thailand: Phetchabun: Thung Salaeng Luang NP, pine forest; Gang Wang Nam Yen, 16°36.284'N, 100°53.128'E, 749 m, 20–21.vi.2007, Pan traps, Pongpitak & Sathit leg., T2056”; 1 male, “Thailand: Phetchabun: Thung Salaeng Luang NP, pine forest; Gang Wang Nam Yen, 16°36.284'N, 100°53.128'E, 749 m, 21–22.vi.2007, Pan traps, Pongpitak & Sathit leg., T2057”; 7 males, “Thailand: Phetchabun: Thung Salaeng Luang NP, pine forest; Gang Wang Nam Yen, 16°36.284'N, 100°53.128'E, 749 m, 22–23.vi.2007, Pan traps, Pongpitak & Sathit leg., T2058”; 1 male, “Thailand: Phetchabun: Thung Salaeng Luang NP, pine forest; Gang Wang Nam Yen, 16°36.284'N, 100°53.128'E, 749 m, 22–29.vi.2007, Malaise trap, Pongpitak & Sathit leg., T2063”; 3 males, “Thailand: Phetchabum, Thung Salaeng Luang NP, Gang Wang Nam Yen, pine forest, 16°35.789N, 100°52.769E, 723 m, Malaise trap, 6–13.vii.2007, Pongpitak Pranee & Sathit leg. T 2068”; 6 males, “Thailand: Phetchabum, Thung Salaeng Luang NP, Gang Wang Nam Yen, 16°36.284N, 100°53.128E, 749 m, Malaise trap, 29.vi–6.vii.2007, Pongpitak Pranee & Sathit leg., T 2069”; 2 males, “Thailand: Phetchabum, Thung Salaeng Luang NP, Gang Wang Nam Yen, pine forest, 16°35.805N, 100°52.286E, 726 m, Malaise trap, 6–13.vii.2007, Pongpitak Pranee & Sathit leg., T 2070”; 3 males, “Thailand: Phetchabun: Thung Salaeng Luang NP: Gang Wang Nam Yen, 750 m, 16°36.587'N, 100°53.395'E; 17–24.v.2007, Pongpitak Pranee & Sathit leg., T2080”; 7 males, “Thailand: Phetchabum, Thung Salaeng Luang NP, Gang Wang Nam Yen, 16°37.178N, 100°.53.504E, 706 m, Malaise trap, 17–24.v.2007, Pongpitak Pranee & Sathit leg., T2081”; 1 males, “Thailand: Phetchabun: Thung Salaeng Luang NP, Gang Wang Nam Yen, 16°36.587'N, 100°53.395'E, 753 m, 24–31.v.2007, Malaise trap, Pongpitak Pranee & Sathit leg., T2083”; 2 males, “Thailand: Nakhon Nayok: Khao Yai NP, Lum Ta Kong View Point, 14°25.82'N, 101°23.754'E, 744 m, 26.iv.2007–2.v.2007, Malaise trap, Pong Sandao leg., T2132”; 5 males, “Thailand: Nakhon Ratchasima: Khao Yai NP, Cobra zone near fire protection office, 14°27.511N, 101°22.408'E, 760 m, 5–12.vi.2007, Malaise trap, Pong Sandao leg., T2223”; 1 male, “Thailand: Nakhon Ratchasima: Khao Yai NP, Cobra zone near fire protection office, 14°28.285N, 101°22.57'E, 751 m, 12–19.vi.2007, Malaise trap, Wirat Sukho leg., T2225”; 1 male, “Thailand: Nakhon Ratchasima: Khao Yai NP, Cobra zone near fire protection office, 14°27.511N, 101°22.408'E, 760 m, 12–19.vi.2007, Malaise trap, Wirat Sukho leg., T2226”; 1 male, “Thailand: Nakhon Nayok: Khao Yai NP, entrance of Hnong Pak Chee Trail, 14°27.115'N, 101°21.951'E, 733 m, 5–6.v.2007, Pan traps, Pong Sandao leg., T2256”; 5 males, “Thailand: Nakhon Nayok: Khao Yai NP, entrance of Hnong Pak Chee Trail, 14°27.115'N, 101°21.951'E, 733 m, 5–12.v.2007, Malaise traps, Wirat Sukho leg., T2264”; 4 males, “Thailand: Nakhon Nayok: Khao Yai NP, entrance of Hnong Pak Chee Trail, 14°27.115'N, 101°21.951'E, 733 m, 12–19.v.2007, Malaise traps, Pong Sandao leg., T2267”; 3 males, “Thailand: Nakhon Nayok: Khao Yai NP, entrance of Hnong Pak Chee Trail, 14°27.119'N, 101°21.482'E, 699 m, 12–19.v.2007, Malaise traps, Wirat Sukho leg., T2268”; 2 males, “Thailand: Nakhon Nayok: Khao Yai NP, entrance of Hnong Pak Chee Trail, 14°27.167'N, 101°21.85'E, 758 m, 19–26.v.2007, Malaise traps, Wirat Sukho leg., T2269”; 2 males, “Thailand: Nakhon Nayok: Khao Yai NP, entrance of Hnong Pak Chee Trail, 14°27.115'N, 101°21.951'E, 733 m, 19–26.v.2007, Malaise traps, Pong Sandao leg., T2270”; 2 males, “Thailand: Nakhon Nayok: Khao Yai NP, entrance of Hnong Pak Chee Trail, 14°27.119'N, 101°21.482'E, 699 m, 19–26.v.2007, Malaise traps, Wirat Sukho leg., T2271”; 1 male, “Thailand: Nakhon Nayok: Khao Yai NP, entrance of Hnong Pak Chee Trail, 14°27.167'N, 101°21.85'E, 758 m, 26.v.2007–2.vi.2007, Malaise traps, Wirat Sukho leg., T2272”; 2 males, “Thailand: Nakhon Nayok: Khao Yai NP, entrance of Hnong Pak Chee Trail, 14°27.115'N, 101°21.951'E, 733 m, 26.v.2007–2.vi.2007, Malaise traps, Pong Sandao leg., T2273”; 1 male, label lost; in QSBG, MNHN, SCAU and CRF, respectively.

#### Etymology.

 The name refers to the similarity of this new species to *Orthogonius chaudoiri*.

#### Distribution.

 Thailand. Known only from the type localities.

### Orthogonius constrictus

Tian, Deuve & Felix sp. n.

urn:lsid:zoobank.org:act:DC47F34A-8BBE-48FB-8D65-14C1AEEF87C6

http://species-id.net/wiki/Orthogonius_constrictus

Figures 10 22

#### Diagnosis.

 Medium sized, labrum sexsetose, nearly straight at apical margin, palpiger asetose, even and odd intervals subequal in width in middle portion, prosternal process bordered at apex; apical margin of abdominal ventrite VII widely and rather deeply emarginate in male; a member of the *Orthogonius longicornis* species group, distinguished by its aedeagus constricted subapically in dorsal view.

**Figures 10–13.**
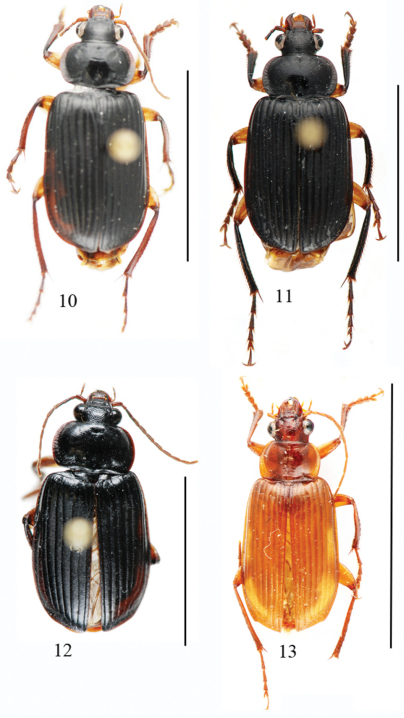
Habitus of *Orthogonius* spp. n. **10** *Orthogonius constrictus* sp. n. holotype **11** *Orthogonius pinophilus* sp. n. holotype **12** *Orthogonius vari* sp. n. holotype **13** *Orthogonius variabilis* sp. n. holotype. Scale bar: 10 mm.

Length: 12.5 mm; width: 5.5 mm. Habitus as in Figure 10.

#### Description.

Dark brown or black, but palpi and femora yellowish brown; trochanters, coxae and lateral pronotal margins reddish brown.

Wrinkles and punctures: surface smooth and impunctate; head and pronotum faintly striate, odd elytral intervals (3, 5 and 7) with an irregular row of fine punctures. Surface strongly shiny.

Microsculptural meshes densely isodiametric on elytra, denser and more transverse on pronotum and head.

Head stout, as long as wide; eyes very large, strongly prominent, frons and vertex moderately convex, frontal impressions small, short, fovea-like, clypeus bisetose, rather even, labrum sexsetose, nearly straight at apical margin; ligula very small and narrow, bisetose at apex; palpi slender, subcylindrical; palpiger asetose, mentum without median tooth, asetose; submentum with one pair of setae. Antennae slender, extended beyond basal 1/4 of elytra, pubescent from apical 2/3 of antennomere 4; antennomeres 3 slightly shorter than 4, antennomeres 4 and 5 subequal in length; antennomeres 1-3 glabrous; antennomeres 4 and 5 distinctly expanded laterally.

Pronotum strongly transverse, PW/PL=1.63, sides evenly rounded, widest at about middle, both basal and fore margins beaded, lateral expanded margins well defined, wide, uneven, smooth and rather flat; both fore and hind angles rounded; disc moderately convex, both transverse impressions not well defined; basal foveae small, middle line distinct.

Elytra ovate (EL/WL=1.55), strongly convex, basal border complete; sides slightly expanded in middle portion, nearly parallel-sided, widest at middle; striae deep, punctate-striate, intervals distinctly convex; intervals 2, 4 and 6 subequal in width, each wider than intervals 1, 3, and 5, respectively, but less than twice as wide; odd intervals with more distinct fine punctures; apex roundly truncate, inner angle broad, without tooth; interval 3 with three well marked setiferous pores (but middle pore absent from left elytron in the holotype and an additional fourth pore present on left elytron in one of the paratypes); interval 7 simple, wide and not carinate, without seta.

Legs moderate, fore tibia with outer angle nearly rectangular, blunt, outer margin faintly serrate; middle and hind coxae smooth and glabrous; middle and hind tibia slender, apex slightly dilated; middle tibia not dilated or curved in middle portion; hind apical tibial spurs very long and sharp; tarsomere 1 much longer than 2, tarsomere 3 slightly longer than 4, tarsomere 4 deeply emarginate at apex (about 1/3 deep as the joint); all tarsal claws strongly pectinate; hind femur moderately dilated, with 2 posterior setae on ventral.

Prosternal process well bordered at apex. Apical margin of abdominal ventrite VII widely and rather deeply emarginate in male.

Male genitalia (Figure 22): Aedeagus elongate, expanded in median portion, sinuate before apex which is more or less bent and pointed at tip; in dorsal view, distinctly constricted before apex, the apical lamella long and slender, 3.4 times as long as wide.

**Female.** Unknown.

#### Remarks.

 This species is a member of the *Orthogonius longicornis* group, but is easily distinguished from other members by its long and slender apical lamella, together with aedeagus more or less constricted before apex in dorsal view.

#### Type material.

Holotype: male, “Thailand: Phetchabum, Thung Salaeng Luang NP, Gang Wang Nam Yen, 16°36.284 N, 100°53.128E, 749 m, Malaise trap, 29.vi–6.vii.2007, Pongpitak Pranee & Sathit leg., T 2069”, in QSBG.

Paratypes: 2 males, ibid, in QSBG and MNHN, respectively.

#### Etymology.

 The species name refers to the more or less constricted base of the apical lamella of aedeagus in dorsal view.

#### Distribution.

 Thailand. Known only from the type locality.

### Orthogonius pinophilus

Tian, Deuve & Felix sp. n.

urn:lsid:zoobank.org:act:B788D24D-357A-494C-849B-BB9720434A09

http://species-id.net/wiki/Orthogonius_pinophilus

Figures 11 23

#### Diagnosis.

 Medium sized, labrum with apical margin straight, prosternal process well bordered at apex, apical margin of abdominal ventrite VII widely emarginate; a member of the *Orthogonius longicornis* species group, and easily recognized by its stout and more or less broad apical lamella.

Length: 13.5 mm; width: 6.0 mm. Habitus as in Figure 11.

#### Description.

Black, clypeus, mouthparts (except tips of mandibles) and palps, antennomere 1, and coxae, trochanters and femora of legs yellowish brown.

Wrinkles and punctures: surface impunctate except elytral intervals 3, 5 and 7 with tiny, and sparse punctures arranged in a row, head wrinkled, pronotum faintly striate.

Microsculptural meshes isodiametric on elytra, faint or more or less irregular on head and pronotum.

Head as long as wide, eyes very large, strongly prominent; frons and vertex convex, frontal impressions faint, clypeus bisetose, even; labrum quadrate, sexsetose, frontal margin straight; mandibles well developed; ligula small, not expanded at apex, bisetose; palpi slender, subcylindrical, maxillary palpomere 3 as long as 4, glabrous; labial palpomere 2 longer than 3, 2-setose in inner margin; labial palpomere 3 sparsely pubescent; palpiger asetose, mentum asetose, submentum with one pair of setae; mentum without median tooth. Antennae, except left antennomeres 1–3 and right antennomere 1 absent.

Pronotum strongly transverse, PW/PL=66/45, strongly convex; sides evenly rounded, widest at about middle, both basal and fore margins beaded, lateral expanded margins well defined, with few transverse striae, slightly reflexed, and uneven, fore and hind angles rounded; fore transverse impression indistinct, hind one faint, basal foveae small, but well marked, middle line distinct.

Elytra ovate, convex, basal border complete, sides slightly expanded, not parallel at middle, striae deep, intervals very convex, intervals subequal in width in middle; apex roundly truncate; interval 3 with three setae, all are well marked; interval 7 not carinate, without seta.

Legs moderate, middle tibia slightly curved in middle, abruptly and slightly dilated at apex; middle coxae glabrous in median portion; hind tibiae slender, slightly dilated only at apex; hind tibial spurs long and sharp; tarsomere 3 much shorter than tarsomere 4 (almost 1:1.5), tarsomere 4 symmetrically and shallowly emarginate at apex (depth of emargination equal about 2/5 length of the joint); femora rather slender, hind femur with two setae posteriorly; all tarsal claws strongly pectinate.

Prosternal process well bordered at apex. Apical margin of abdominal ventrite VII widely emarginate.

Male genitalia (Figure 23): Median lobe of aedeagus moderately stout for the group, slightly dilated in middle portion, gradually constricted towards apex in lateral view; in dorsal view apical lamella stout and somewhat expanded at tip.

**Female.** Unknown.

#### Remarks.

 This species is a member of the *Orthogonius longicornis* species group, but is easily separated from other members by its stout apical lamella of aedeagus.

#### Material examined.

 Holotype: male, “Thailand: Phetchabum, Thung Salaeng Luang NP, Gang Wang Nam Yen, pine forest, 16°36.284N, 100°53.128E, 749 m, Malaise trap, 29.vi–6.vii.2007, Pongpitak Pranee & Sathit leg., T 2066”, in QSBG.

#### Etymology.

 The name refers to the fact that the holotype of this new species was collected in pine forest.

#### Distribution.

 Thailand. Know only from the type locality.

### Orthogonius vari

Tian, Deuve & Felix sp. n.

urn:lsid:zoobank.org:act:C88DF58F-C106-45F6-BDEA-57AF91BF66B5

http://species-id.net/wiki/Orthogonius_vari

Figures 12 24

#### Diagnosis.

 A stout and broad species; eyes very large and prominent, pronotum and elytra strongly convex; labrum straight at front; mentum asetose; lateral expanded margin of pronotum tapered from base to front, not reflexed; elytra well bordered at base, apex roundly truncate, inner angle broad; interval 3 with three setiferous pores, interval 7 normal; prosternal process well bordered at apex; ventrite VII in male distinctly emarginate; fore tibia with outer angle nearly rectangular, blunt, and not protruded, outer margin not serrate; middle and hind tibiae slender; hind tibial spur very long and sharp, hind tarsomere 3 much longer than 4, tarsomere 4 shallowly emarginate; all tarsal claws very strongly pectinate; femora moderately dilated; hind femur with two posterior setae.

Length: 13.0-14.0 mm; width: 6.3-6.5 mm. Habitus as in Figure 12.

#### Description.

Black on upper and lower surfaces, except margin of pronotum, antennae (1-2 much lighter than other antennomeres), palpi, and labrum brown, coxae, trochanters and femora yellowish, tibiae and tarsi dark brown.

Surface smooth and impunctate; head intricately striate, pronotum very finely striate; odd elytral intervals (3, 5, 7) with distinct fine punctures in an irregularly row. Surface strongly shiny. Microsculptural meshes densely isodiametric, clear on elytra, but faint on pronotum and head.

Head stout, wider than long; HW/HL=1.1, eyes very large, strongly prominent, frons and vertex moderately convex, frontal impressions small, short, fovea-like, clypeus bisetose, rather even, labrum sexsetose, straight at apical margin; ligula very small and narrow, bisetose at apex; palpi slender, subcylindrical, normal; palpiger asetose, mentum without median tooth, asetose, submentum with one pair of setae. Antennae slender, extended to basal 1/3 of elytra, pubescent from apical 2/3 of antennomere 4; antennomere 3 as long as 4, both shorter than antennomere 1 and slightly longer than 5; antennomeres 1-3 glabrous; antennomere 4 distinctly expanded laterally.

Pronotum strongly transverse, PW/PL=1.57, sides evenly rounded, widest at about middle, both basal and fore margins beaded, lateral expanded margins well defined, flat and distinctly tapered from base to front, wide and smooth; both fore and hind angles rounded; disc strongly convex, fore transverse impression indistinct, basal transverse impression moderate, basal foveae distinct and deep.

Elytra broadly ovate (EL/WL=1.46), strongly convex, basal border complete; sides slightly expanded in middle portion, slightly parallel-sided, widest slightly behind middle; striae deep, punctate-striate; intervals slightly convex, subequal in width; odd intervals with more distinct fine punctures in an irregular row; apex roundly truncate, inner angle broad; interval 3 with three setiferous pores, near striae 3, 2 and 2, respectively, and well marked; interval 7 normal, wide and not carinate, without setiferous pore.

Middle and hind coxae smooth and glabrous. Legs moderate, fore tibiae with outer angle nearly rectangular, blunt, outer margin not serrate; middle and hind tibiae slender, apex slightly dilated, apical spurs very long and sharp; tarsomere 3 much longer than 4, tarsomere 4 shallowly emarginate at apex; all tarsal claws strongly pectinate; hind femur with 2 posterior setae on ventral.

Prosternal process well bordered at apex. Apical margin of abdominal ventrite VII widely but shallowly emarginate in male.

Male genitalia (Figure 24): Median lobe long and distinctly expanded in median portion, upper margin abruptly sinuate, apex gradually tapered; ventral margin sinuate, dorsal opening long and wide; the apical lamella quite elongate, two times as long as wide, and tip rounded, and nearly parallel-sided.

**Female.** Unknown.

#### Remarks.

 This species is similar to *Orthogonius kirirom* Tian & Deuve, 2008, but is easily distinguished from the latter by its stouter body, elytral inner angle broad, hind femur slightly more dilated, and aedeagus more elongate, with apical lamella more slender and almost parallel-sided.

#### Material examined.

Holotype, male, “Coll. I. R. Sc. N. B. / Cambodia, Siem Reap Prov., Angkor Preah Kahm, Malaise Trap, 28/III-05/IV-2006. leg. I. Var”; in IRSNB.

Paratypes: 1 male, “Thailand: Ubon Ratchathani: Pha Taem NP, Wild flower field 1, 15°27.336'N, 105°34.87'E, 232 m, 23–30.v.2007, Malaise trap, Sorawit Mingman leg., T2195”; 1 male, “Thailand: Phetchabun: Thung Salaeng Luang NP, pine forest; Gang Wang Nam Yen, 16°36.284'N, 100°53.128'E, 749 m, 20–21.vi.2007, Pan traps, Pongpitak & Sathit leg., T2056”; 1 male, “Thailand: Phetchabum, Thung Salaeng Luang NP, Gang Wang Nam Yen, 16.37.178 N, 100.53.504 E, 706 m, Malaise trap, 17–24.v. 2007, Pongpitak Pranee & Sathit leg. T2081”; in QSBG and MNHN respectively.

#### Etymology.

 This new species is named in honor of Mr I. Var, the collector of the holotype.

#### Distribution.

 Cambodia and Thailand.

### Orthogonius kirirom

Tian & Deuve, 2008

http://species-id.net/wiki/Orthogonius_kirirom

#### Material examined.

1 male, “Thailand: Ubon Ratchathani: Pha Taem NP, wild flower field, 15°27.336'N, 105°34.87'E, 232 m, 2–9.v.2007, Malaise trap, Sorawit Mingman leg., T2186”, in QSBG; 1 male, “Thailand: Phetchabun: Thung Salaeng Luang NP: Gang Wang Nam Yen, 750 m, 16°36.587'N, 100°53.395'E; 17–24.v.2007, Pongpitak Pranee & Sathit leg., T2080”, in QSBG.

#### Distribution.

 Cambodia and Thailand. This species is recorded from Thailand here for the first time.

### Orthogonius leoeinsis

Tian & Deuve, 2006

http://species-id.net/wiki/Orthogonius_leoeinsis

#### Material examined.

 1 male, “Thailand: Ubon Ratchathani: Pha Taem NP, wild flower field, 15°27.336'N, 105°34.87'E, 232 m, 2–9.v.2007, Malaise trap, Sorawit Mingman leg., T2186”, in QSBG.

#### Remarks.

 Head punctate, pronotum glabrous; the aedeagus of our specimen is slightly wider than that of the type specimen.

#### Distribution.

 Thailand.

### Orthogonius thailandensis

Tian & Deuve, 2006

http://species-id.net/wiki/Orthogonius_thailandensis

#### Material examined.

 1 male, “Thailand: Phetchabum, Thung Salaeng Luang NP, Gang Wang Nam Yen, 16°36.284N, 100°53.128E, 749 m, Malaise trap, 29.vi–6.vii.2007, Pongpitak Pranee & Sathit leg., T 2069”, in QSBG.

#### Remarks.

 Our specimen is a smaller individual, and slightly stouter than the holotype specimen; length 8.5 mm, width 3.7 mm;

#### Distribution.

Thailand.

### Orthogonius pseudolongicornis

Tian & Deuve, 2006

http://species-id.net/wiki/Orthogonius_pseudolongicornis

#### Material examined.

 2 males, “Thailand: Phetchabum, Thung Salaeng Luang NP, Gang Wang Nam Yen, pine forest, 16°35.789N, 100°52.769E, 723 m, Malaise trap, 6–13.vii.2007, Pongpitak Pranee & Sathit leg., T2068”; 1 male, “Thailand: Phetchabun: Thung Salaeng Luang NP, Gang Wang Nam Yen, 16°37.178'N, 100°53.504'E, 706 m, 24–31.v.2007, Malaise trap, Pongpitak Pranee & Sathit leg., T2084”, in QSBG and MNHN.

#### Remarks.

 In one of the specimens from sample T2068, the ligula is very thin and narrow, 6-setose at apex (rather than 4-setae as Ron Felix's noted label), but all other characters are typical for the species. Therefore, we treat it as an abnormal individual.

#### Distribution.

 Myanmar, Vietnam, Cambodia and Thailand.

### Orthogonius longicornis

Chaudoir, 1871

http://species-id.net/wiki/Orthogonius_longicornis

#### Material examined.

 2 males, “Thailand: Phetchabun: Thung Salaeng Luang NP, pine forest; Gang Wang Nam Yen, 16°36.284'N, 100°53.128'E, 749 m, 16–17.vi.2007, Pan traps, Pongpitak & Sathit leg., T2052”; 1 male, “Thailand: Phetchabun: Thung Salaeng Luang NP, pine forest; Gang Wang Nam Yen, 16°36.284'N, 100°53.128'E, 749 m, 20–21.vi.2007, Pan traps, Pongpitak & Sathit leg., T2056”; 1 male, “Thailand: Sakon Nakhon: Phu Phan NP, Kam Hom waterfall at Haew Sin Chai, 17°7.415'N, 104°1.179'E, 347 m, 16.ix.2006–22.ix.2006, Malaise trap, Winlon Khongnara leg., T616”; 1 male, “Thailand: Loei: Phu Kradueng NP, dry dipterocarp forest at Loei forest unit 2 (E-lerd), 16°56.651'N, 101°48.903'E, 273 m, 18.ix.2006–25.ix.2006, Malaise trap, Sutin Glong-Lasae leg., T952”; 1 male, “Thailand: Loei: Phu Kradueng NP, Bamboo forest at Lam Huay Taad at Loei, forest unit 2 (E-lerd), 16°56.565'N, 101°48.896'E, 273 m, 11.ix.2006–18.ix.2006, Malaise trap, Sutin Glong-Lasae leg., T948”; 1 male, “Thailand: Loei: Phu Kradueng NP, Koke Hin Ngam, 16°51.958'N, 101°50.668'E, 280 m, 9.viii.2006–16.viii.2006, Malaise trap, Sutin Khonglasae, T482”, in QSBG, MNHN, SCAU and CRF, respectively.

#### Distribution.

 Thailand.

### Orthogonius nahaeo

Tian & Deuve, 2006

http://species-id.net/wiki/Orthogonius_nahaeo

#### Material examined.

 3 males, “Thailand: Ubon Ratchathani: Pha Taem NP, wild flower field, 15°27.336'N, 105°34.87'E, 232 m, 2–9.v.2007, Malaise trap, Sorawit Mingman leg., T2186”; 1 male, “Thailand: Phetchabun: Thung Salaeng Luang NP, Gang Wang Nam Yen, 16°36.587'N, 100°53.395'E, 753 m, 31.v.2007–7.vi.2007, Malaise trap, Pongpitak Pranee & Sathit leg., T2086”. in QSBG, MNHN and SCAU, respectively.

#### Remarks.

 In general, members of *Orthogonius nahaeo* have no seta on the mentum, but in one specimen collected in sample T2186, the mentum has a short seta (compared to setae on the submentum) on the right side.

#### Distribution.

 Thailand.

### Orthogonius siamensis

Tian & Deuve, 2006

http://species-id.net/wiki/Orthogonius_siamensis

#### Material examined.

 1 male, “Thailand: Phetchabum, Thung Salaeng Luang NP, Gang Wang Nam Yen, 16°36.284N, 100°53.128E, 749 m, Malaise trap, 29.vi-6.vii.2007, Pongpitak Pranee & Sathit leg., T 2069”, in QSBG.

#### Distribution.

 Thailand.

### Orthogonius mouhoti

Chaudoir, 1871

http://species-id.net/wiki/Orthogonius_mouhoti

#### Material examined.

 2 males, “Thailand: Phetchabun: Thung Salaeng Luang NP, Gang Wang Nam Yen, 16°37.178'N, 100°53.504'E, 706 m, 24–31.v.2007, Malaise trap, Pongpitak Pranee & Sathit leg., T2084”; 1 male, “Thailand: Chaiyaphum: Pa Hin Ngam NP, Ecotone between mix deciduous/dry dipterocarp, 15°38.1'N, 101°23.857'E, 700 m, 5.viii.2006–11.viii.2006, Malaise trap, Katae Sa-Nog & Buakaw Adnafai leg., T440”; 1 male, “Thailand: Loei: Phu Kradueng NP, Huay Ta Hack, 16°51.958'N, 101°50.668'E, 280 m, 30.viii.2006–6.ix.2006, Malaise trap, Sutin Khonglasae leg., T491”; 1 male, “Thailand: Ubon Ratchathani: Pha Taem NP, Don Huay Sa-nhom, 15°27.435'N, 105°34.838'E, 238 m. 9–16.v.2007, Malaise trap, Sorawit Mingman leg., T2187”; 1 male, “Thailand: Chaiyaphum: Tat Tone NP, Lam Pa Tao, dry evergreen forest head water, 15°58.486'N, 102°2.239'E, 270 m, 5.viii.2006–12.viii.2006, Malaise trap, Tawit Jaruphan & Orawan Budsawong leg., T546”; 1 male, “Thailand: Loei: Phu Kradueng NP, Koke Hin Ngam, 16°51.958'N, 101°50.668'E, 280 m, 9.viii.2006–16.viii.2006, Malaise trap, Sutin Khonglasae, T482”; 1 male, “Thailand: Phetchabun: Thung Salaeng Luang NP, Gang Wang Nam Yen, 16°37.178'N, 100°53.504'E, 706 m, 24–31.v.2007, Malaise trap, Pongpitak Pranee & Sathit leg., T2084”; 1 male, “Thailand: Ubon Ratchathani: Pha Taem NP, Wild flower field 1, 15°27.336'N, 105°34.87'E, 232 m, 23–30.v.2007, Malaise trap, Sorawit Mingman leg., T2195”, in QSBG, MNHN, SCAU and CRF, respectively.

#### Distribution.

 Laos and Thailand. This species is here first recorded from Thailand.

### Orthogonius variabilis

Tian, Deuve & Felix sp. n.

urn:lsid:zoobank.org:act:FE855CF8-60A2-4705-9F5D-C5A4909A9AA5

http://species-id.net/wiki/Orthogonius_variabilis

Figures 13 15 25

#### Diagnosis.

 Medium to quite small sized, ligula quadrisetose or sexsetose (in 4 paratypes), aedeagus somewhat similar to that of *Orthogonius perakensis* Tian & Deuve, 2006; elytral interval 3 with two setiferous pores (middle pore absent); elytra obliquely and sinuately truncate, with apical inner angles acute and sharp.

Length: 9.0–13.5 mm; width: 4.0–5.0 mm. Habitus as in Figure 13–15.

#### Description.

Body dark brown to yellow (That means the coloration is variable for some species of Orthogoniini, and the size too, not only shapes, but legs, pronotum, elytra, head and so on as well).

Body with varied coloration: from yellowish to black.

Upper surface smooth and glabrous, impunctate (but punctate in one paratype), elytral intervals each with an irregular row of tiny punctures along median portion; moderately shiny.

Microsculptural meshes densely isodiametric on elytra, irregularly and densely on head and pronotum.

Head stout, slightly wider than long, eyes very large and strongly prominent; frons and vertex moderately convex, frontal impressions small and fovea-like, clypeus bisetose, surface with a transverse impression and a median fovea near base; labrum sexsetose, frontal margin almost straight; ligula short, quadrisetose (sexsetose in a few individuals) at apex; palpi slender, subcylindrical, maxillary palpomere 4 longer than 3, palpomere 3 glabrous, except several setae at apex; maxillary palpomere 4 glabrous with very short setae; labial palpomere 3 slightly shorter than palpomere 2, palpomere 3 with a few setae at base; labial palpomere 2 bisetose in inner margin, and with two or three additional setae at subapex and apex; palpiger asetose, mentum and submentum each with one pair of setae; mentum without median tooth. Antennae moderate, extended to middle of elytra; pubescent from basal 1/3 of antennomere 4; antennomere 3 slightly shorter than antennomere 4.

Pronotum transverse, widest at about middle, PW/PL=1.17–1.22, disc slightly and evenly convex, both angles broadly rounded, both basal and fore margins beaded, lateral expanded margins wide, rather flat or somewhat reflexed; fore and hind transverse impressions distinct, basal foveae well-marked.

Elytra elongate ovate, EL/EW=1.7; moderately convex, basal border complete, shoulders broadly square; sides more or less parallel at middle, widest at about middle; striae deep, punctate-striate; intervals moderately convex, and subequal in width in middle; apex quite narrowly and obliquely truncate, outer angle well marked, inner angle sharp and denticulate; interval 3 with two setiferous pores (the middle pore absent); interval 7 simple.

Legs slender. Fore tibiae with apical outer angle obtuse; outer margin distinctly serrate; middle and hind coxae smooth and glabrous; middle tibiae evenly curved, gradually dilated towards apex; hind tibiae slender, apical spurs short and sharp, tarsomere 1 slightly longer than 2, tarsomere 3 distinctly longer than 4, tarsomere 4 bilobed; fore tarsi much wider than middle and hind ones; all tarsal claws strongly pectinate.

Prosternal process unbordered at apex. Apical margin of abdominal ventrite VII of male narrowly but distinctly emarginate at apical margin.

Male genitalia (Figure 25): the median lobe of the aedeagus somewhat stout, slightly or evenly expanded at middle portion; dorsal opening wide and long; in dorsal view apical lamella very short, , broadly pointed at apex.

**Figures 14–15.**
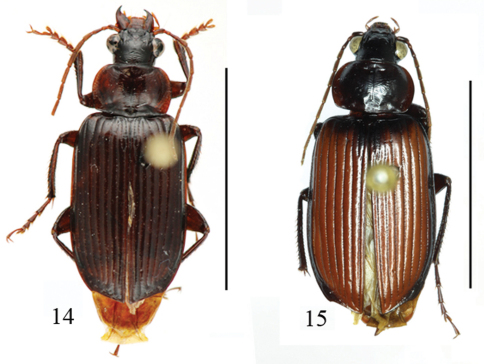
Habitus of *Orthogonius variabilis* sp. n. paratypes. Scale bar: 10 mm.

**Figures 16–18.**
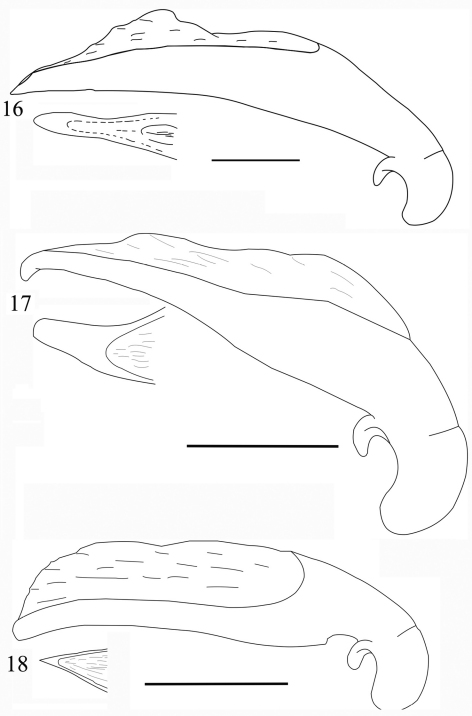
Aedeagus of *Orthogonius* spp. n. (lateral view, and apex in dorsal view) **16** *Orthogonius taghavianae* sp. n. holotype **17** *Orthogonius coomanioides* sp. n. holotype **18** *Orthogonius similaris* sp. n. holotype. Scale bar: 1 mm.

**Figures 19–22.**
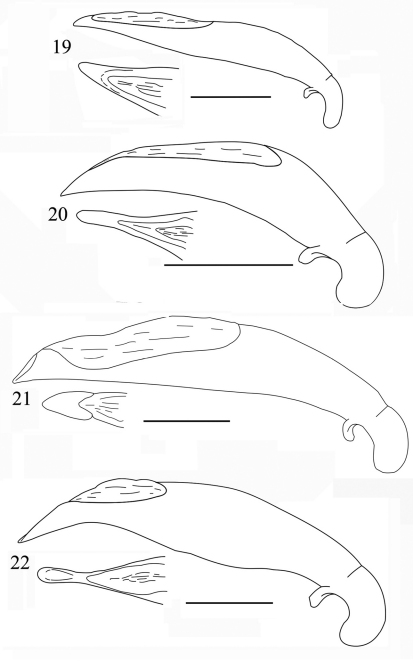
Aedeagus of *Orthogonius* spp. n. (lateral view, and apex in dorsal view) **19** *Orthogonius setosopalpiger* sp. n. holotype **20** *Orthogonius gracililamella* sp. n. holotype **21** *Orthogonius pseudochaudoiri* sp. n. holotype **22** *Orthogonius constrictus* sp. n. holotype. Scale bar: 1 mm.

**Figures 23–25.**
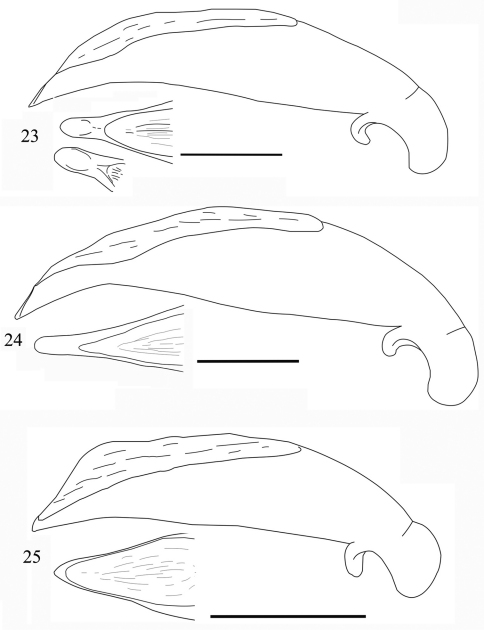
Aedeagus of *Orthogonius* spp. n. (lateral view, and apex in dorsal view) **23** *Orthogonius pinophilus* sp. n. holotype **24** *Orthogonius vari* n. sp. holotype **25** *Orthogonius variabilis* sp. n. holotype. Scale bar: 1 mm.

#### Remarks.

 This species differs from the Perakean species, *Orthogonius perakensis* Tian & Deuve, 2006, by its slender and flat body; and is easily separated from *Orthogonius perroti* Tian & Deuve, 2006 by the shape of its elytral apex.

#### Variability.

 To treat this species is somewhat a challenge, because of the variability in several important characters such as coloration, shape of pronotum, elytral apex, aedeagus and middle tibia, and seta number on ligula. Several species might be “recognized” if there were only a few individuals available. Fortunately the large series of the specimens make it possible to realize the complicated variations of this species. The variations appeared in the following aspects: (1) sized: 9.0–13.5 mm; (2) coloration: from pure yellowish (4 ex), brown (24 ex), dark brown (13 ex), then to black (10 ex). Among Chinese specimens, five are bicoloured on the elytra (Fig. 15); (3) generally the apex of elytra of this species distinctly obliquely truncate, but in one male paratype the outer angle of apical elytra rounded and less sinuate, and inner angle less pointed; (4) pronotum: narrow to wide, and intermediate shapes, occurring in specimens of different size and coloration; (5) middle tibia: slightly curved (majority) or distinctly curved (11 ex); slightly dilated (29 ex) or strongly dilated in median portion; (6) aedeagus: stouter (6 ex) or a little more elongate at apex in dorsal view; (7) ligula: generally quadrisetose, only sexsetose in three specimens in Thianland species (but on the contrary, sexsetose in most Chinese specimens); and (8) punctures: generally impunctate, but one specimen distinctly punctate on vertex of the head.

According to Chaudoir (1871), presence of a sexsetose ligula is one main character for the genus *Hexachaetus* Chaudoir. Nonetheless, we treat this species as a member of *Orthogonius* considering that the number of setae on the ligula is variable.

#### Material examined.

 Holotype: male, “Thailand: Phetchabun: Thung Salaeng Luang NP, Gang Wang Nam Yen, 16°37.531'N, 100°53.745'E, 721 m, 24–31.v.2007, Malaise trap, Pongpitak Pranee & Sathit leg., T2085”, in QSBG.

#### Paratypes.

**Thailand.** 2 males, ibid; 1 male, “Thailand: Phitsanulok: Thung Salaeng Luang NP, moist evergreen, 16°50.641'N, 100°52.894'E, 557 m, 11.viii.2006–18.viii.2006, Malaise trap, Pongpitak Pranee leg., T566”; 1 male, “Thailand: Phetchabum, Thung Salaeng Luang NP, Gang Wang Nam Yen, pine forest, 16°36.284N, 100°53.128E, 749 m, Malaise trap, 29.vi–6.vii.2007, Pongpitak Pranee & Sathit leg., T 2066”; 15 males, “Thailand: Nakhon Nayok: Khao Yai NP, entrance of Hnong Pak Chee Trail, 14°27.167'N, 101°21.85'E, 758 m, 26.v.2007–2.vi.2007, Malaise traps, Wirat Sukho leg., T2272”; 1 male, “Thailand: Phetchabum, Thung Salaeng Luang NP, Gang Wang Nam Yen, pine forest, 16°35.789N, 100°52.769E, 723 m, Malaise trap, 6–13.vii.2007, Pongpitak Pranee & Sathit leg., T 2068”; 2 males, “Thailand: Nakhon Nayok: Khao Yai NP, entrance of Hnong Pak Chee Trail, 14°27.167'N, 101°21.85'E, 758 m, 5–12.v.2007, Malaise traps, Pong Sandao leg., T2263”; 3 males, “Thailand: Nakhon Ratchasima: Khao Yai NP, Cobra zone near fire protection office, 14°28.285'N, 101°22.57'E, 751 m, 5–12.vi.2007, Malaise trap, Wirat Sukho leg., T2222”; 1 male, “Thailand: Phetchabun: Nam Nao NP, Checkpoint, 16°43.695'N, 101°33.797'E, 921 m, 27.ii.2007–1.iii.2007, Litter sample, Noopean Hongyothi & Leng Janteab leg., T2275”; 7 males, “Thailand: Nakhon Nayok: Khao Yai NP, entrance of Hnong Pak Chee Trail, 14°27.167'N, 101°21.85'E, 758 m, 19–26.v.2007, Malaise traps, Wirat Sukho leg., T2269”; 1 male, “Thailand: Nakhon Ratchasima: Khao Yai NP, Cobra zone near fire protection office, 14°28.285N, 101°22.57'E, 751 m, 12–19.vi.2007, Malaise trap, Wirat Sukho leg., T2225”; 2 males, “Thailand: Nakhon Ratchasima: Khao Yai NP, Cobra zone near fire protection office, 14°28.524N, 101°22.928'E, 757 m, 19–26.vi.2007, Malaise trap, Wirat Sukho leg., T2227”; 3 males, “Thailand: Nakhon Nayok: Khao Yai NP, entrance of Hnong Pak Chee Trail, 14°27.119'N, 101°21.482'E, 699 m, 19–26.v.2007, Malaise traps, Wirat Sukho leg., T2271”; 1 male, “Thailand: Nakhon Ratchasima: Khao Yai NP, Cobra zone near fire protection office, 14°28.524N, 101°22.928'E, 757 m, 12–19.vi.2007, Malaise trap, Pong Sandao leg., T2224”; 1 male, “Thailand: Nakhon Ratchasima: Khao Yai NP, Cobra zone near fire protection office, 14°28.524'N, 101°22.928'E, 757 m, 5–12.vi.2007, Malaise trap, Pong Sandao leg., T2221”; 2 males, “Thailand: Nakhon Ratchasima: Khao Yai NP, Elephant Trail near fire protection office, 14°28.285'N, 101°22.57'E, 751 m, 26.vi.2007–2.vii.2007, Malaise trap, Wirat Sukho leg., T2231”; 1 male, “Thailand: Phetchabun: Thung Salaeng Luang NP, pine forest; Gang Wang Nam Yen, 16°35.805'N, 100°52.286'E, 726 m, 22–29.vi.2007, Malaise trap, Pongpitak & Sathit leg., T2064”; 2 males, “Thailand: Phetchabun: Thung Salaeng Luang NP, pine forest; Gang Wang Nam Yen, 16°36.284'N, 100°53.128'E, 749 m, 18–19.vi.2007, Pan traps, Pongpitak & Sathit leg., T2054”; 2 males, “Thailand: Phetchabun: Thung Salaeng Luang NP, pine forest; Gang Wang Nam Yen, 16°36.284'N, 100°53.128'E, 749 m, 20–21.vi.2007, Pan traps, Pongpitak & Sathit leg., T2056”; 1 male, “Thailand: Phetchabun: Thung Salaeng Luang NP: Gang Wang Nam Yen, 750 m, 16°36.587'N, 100°53.395'E; 17–24.v.2007, Pongpitak Pranee & Sathit leg., T2080”; 1 male, “Thailand: Phetchabum, Thung Salaeng Luang NP, Gang Wang Nam Yen, 16°37.178'N, 100°.53.504'E, 706 m, Malaise trap, 17–24.v.2007, Pongpitak Pranee & Sathit leg., T2081”, deposited in QSBG, MNHN, SCAU and CRF, respectively. **China.** 5 males and 5 females, “China: Yunnan, Jinghong, Banna NR, Mandian (forest), 22°12.961'N, 100°66.612'E, 746 m, Flying-stop trap, 26.v.2009, Meng Lingzeng leg.”; 6 males and 18 females, ibid, 06.vi.2009; 1 male and 1 female, ibid, 26.vi.2009; 4 males and 3 females, ibid, 16.vi.2009; 3 females, ibid, pitfall trap 16.vi.2009; 1 male, ibid, rubber forest, 26.vi.2009; 1 male and 3 females, ibid, 16.v.2009; 1 female, ibid, 22°13.059'N, 100°66.817'E, 753 m, 16.vi.2009; 1 female, ibid, 26.vi.2009; 1 female, ibid, 26.v.2009; 1 female, ibid, 16.v.2009; 1 female, ibid, 06.vi.2009; 2 males and 1 female, “China: Yunnan, Jinghong, Banna NR, Anmaxinzhai (forest), 22°19.577'N, 100°64.532'E, 772 m, Flying-stop trap, 16.vi.2009, Meng Lingzeng leg.”; 1 male and 1 female, ibid, 26.vi.2009; 2 males, ibid, 06.vi.2009; 1 male, ibid, 6.vi.2009; 1 male, “China: Yunnan, Jinghong, Banna NR, Guomengshan (paddy-field), 22°24.527'N, 100°60.380'E, 1110 m, Malaise, 26.v.2009, Meng Lingzeng leg.”; 1 female, ibid, 26.vi.2009; 1 male, ibid, forest, 22°24.644'N, 100°60.616'E, 1114 m, 26.vi.2009; 1 female, ibid, 22°24.526'N, 100°60.411'E, 1107 m, 06.vi.2009; 1 male and 1 female, ibid, 6.vi.2009; 1 female, ibid, pitfall trap, 06.vi.2009; 2 males and 1 female, “China: Yunnan, Jinghong, Banna NR, Mandian (rubber forest), 22°13.059'N, 100°66.817'E, 753 m, Flying-stop trap, 16.v.2009, Meng Lingzeng leg.”; 4 males and 1 female, “China: Yunnan, Jinghong, Banna NR, Danuoyou (waste land), 22°20.699'N, 100°63.761'E, 770 m, Malaise, 16.v.2009, Meng Lingzeng leg.”; 1 female, ibid, 6.vi.2009; 1 male and 1 female, “China: Yunnan, Jinghong, Banna NR, Naban tea garden (waste land), 22°15.857'N, 100°66.529'E, 709 m, Malaise, 26.v.2009, Meng Lingzeng leg.”; 1 female, ibid, 6.vi.2009; 1 female, ibid, 16.v.2009; 1 male, ibid, 22°13.091'N, 100°66.861'E, 689 m, 26.v.2009; 1 male, ibid, rubber forest, 22°15.843'N, 100°66.487'E, 732 m, Yellow-pot, 26.v.2009; 1 female, ibid, Malaise; 1 male and 3 females, ibid, forest, 22°15.810'N, 100°66.543'E, 729 m, Flying-stop trap; 1 female, ibid, 26.v.2009; 1 male, ibid, Malaise; 1 male, ibid, 16.v.2009; 2 females, ibid, 22°15.843'N, 100°66.487'E, 732 m, 26.vi.2009;1 female, “China: Yunnan, Jinghong, Banna NR, Jinghong Farm, rubber forest, 22°10.607'N, 100°68.500'E, 759 m, Malaise, 16.v.2009, Meng Lingzeng leg.”; all are deposited in IOZ, except 5 males and 5 females in SCAU.

#### Etymology.

 The species name, “*variabilis*”, means changeable and refers to the varied characters of this new species.

#### Distribution.

 Thailand and China.

##### Unidentified materials

There are 27 specimens still not identified. All of them are females except one male (from Khao Pu-Khao Ya National Park, Trang), from which the aedeagus has been lost. Without reference to male genital characteristics, it is almost impossible to identify the *Orthogonius* species which belonging to either *Orthogonius longicornis* species group (viz. *Orthogonius mouhoti*, *Orthogonius thaicus*, *Orthogonius pseudochaudoiri*, *Orthogonius nahaeo*, *Orthogonius loeicus*, *Orthogonius constrictus*, *Orthogonius vari*, *Orthogonius kirirom*, *Orthogonius pinophilus*, *Orthogonius gracililamella*, *Orthogonius longicornis*, *Orthogonius pseudolongicornis* etc.) or *Orthogonius alternans* species group (viz. O. *taghavianae*, *Orthogonius paris*, *Orthogonius thaiensis*, *Orthogonius pangi*, *Orthogonius huananoides* etc.) in Thailand and its adjacent countries.

### A provisional key to species of Orthogonius in Thailand

**Table:** 

1	Ligula quadrisetose or sexsetose at apex	2
–	Ligula bisetose at apex	3
2	Ligula quadrisetose in all individuals, surface densely punctate, elytra roundly truncate at apex	*Orthogonius similaris* sp. n.
–	Ligula quadrisetose or sexsetose, surface impunctate, elytra obliquely and sinuately truncate, with apical inner angles acute and very sharp	*Orthogonius variabilis* sp. n.
3	Palpiger unisetose at base	4
–	Palpiger asetose at base	5
4	Small (11 mm in length), hind femur quadrisetose posteriorly	*Orthogonius setosopalpiger* sp. n.
–	Larger (14-16 mm in length), hind femur sexsetose posteriorly	*Orthogonius grootaerti* Tian & Deuve, 2006
5	Even elytral intervals distinctly and coarsely punctate, much wider than odd intervals	6
–	Even elytral intervals glabrous, as wide as odd intervals	11
6	Median lobe of aedeagus quite straight in profile, symmetrically constricted at subapex in dorsal aspect (Fig. 16)	*Orthogonius taghavianae* sp. n.
–	Median lobe of aedeagus more or less bent in profile, not symmetrically constricted at subapex	7
7	Even elytral intervals very wide, more than twice as wide as odd intervals	8
–	Even elytral intervals normal, less than twice width of odd intervals	*Orthogonius coomanioides* sp. n.
8	Midcoxa setose, 3^rd^ elytral interval with at least two setiferous pores	9
–	Midcoxa glabrous, 3^rd^ elytral interval with only one setiferous pore	*Orthogonius paris* Tian & Deuve, 2006
9	Apical lamella of aedeagus short, as long as wide (Fig. 26)	*Orthogonius thaiensis* Tian & Deuve, 2006
–	Apical lamella of aedeagus longer	10
10	Median lobe of aedeagus less arcuate ventrally, apical lamella broader (Fig. 27)	*Orthogonius pangi* Tian & Deuve, 2006
–	Median lobe of aedeagus more arcuate ventrally, apical lamella narrower (Fig. 28)	*Orthogonius huananoides* Tian & Deuve, 2006
11	7^th^ elytral interval carinate at basal portion	*Orthogonius morvani* Tian & Deuve, 2003
–	7^th^ elytral interval not carinate	12
12	Mentum with a pair of setae	13
–	Mentum asetose	17
13	Labrum straight at frontal margin	*Orthogonius mouhoti* Chaudoir, 1871
–	Labrum emarginate at frontal margin	14
14	Clypeus quadrisetose	*Orthogonius thaicus* Tian & Deuve, 2003
–	Clypeus bisetose	15
15	Median lobe of aedeagus with apex arrowhead-shaped in dorsal view (Fig. 21)	*Orthogonius pseudochaudoiri* sp. n.
–	Median lobe of aedeagus with apex not arrowhead-shaped	16
16	Apical lamella of aedeagus shorter (Fig. 29)	*Orthogonius nahaeo* Tian & Deuve, 2006
–	Apical lamella of aedeagus longer (Fig. 30)	*Orthogonius loeicus* Tian & Deuve, 2006
17	Labrum straight at frontal margin	18
–	Labrum either convex or emarginate at frontal margin	25
18	Median lobe of aedeagus notched at apical tip (Fig. 31)	*Orthogonius loeiensis* Tian & Deuve, 2006
–	Median lobe of aedeagus not notched	19
19	Median lobe of aedeagus constricted at sub-apex (Fig. 22)	*Orthogonius constrictus* sp. n.
–	Median lobe of aedeagus not constricted	20
20	Apical lamella of aedeagus elongate, almost twice as long as wide	21
–	Apical lamella of aedeagus stout, clearly less twice as long as wide	23
21	Apical lamella of aedeagus nearly parallel-sided (Fig. 24)	*Orthogonius vari* sp. n.
–	Apical lamella of aedeagus gradually narrowed towards apex	22
22	Median lobe of aedeagus stout (Fig. 32)	*Orthogonius kirirom* Tian & Deuve, 2008
–	Median lobe of aedeagus elongate (Fig. 36)	*Orthogonius pseudolongicornis* Tian & Deuve, 2006
23	Median lobe of aedeagus broad at apex in dorsal view (Fig. 23)	24
–	Median lobe of aedeagus narrowed at apex in dorsal view (Fig. 20)	25
24	4^th^ hind tarsomere shallowly emarginate at apex	*Orthogonius kubani* Tian & Deuve, 2006
–	4^th^ hind tarsomere deeply emarginate at apex	*Orthogonius pinophilus* sp. n.
25	Median lobe of aedeagus longer, distinctly sinuate at sub-apex (Fig. 33)	*Orthogonius pachlatkoi* Tian & Deuve, 2006
–	Median lobe of aedeagus shorter, not sinuate at sub-apex (Fig. 34)	*Orthogonius siamensis* Tian & Deuve, 2006
26	Labrum emarginate at frontal margin	27
–	Labrum convex at frontal margin	29
27	Apical lamella of aedeagus narrow and elongate	28
–	Apical lamella of aedeagus stout and wide (Fig. 35)	*Orthogonius chiangensis* Tian & Deuve, 2006
28	Apical lamella of aedeagus longer, nearly parallel-sided (Fig. 20)	*Orthogonius gracililamella* sp. n.
–	Apical lamella of aedeagus shorter, gradually constricted towards apex (Fig. 37)	*Orthogonius longicornis* Chaudoir, 1871
29	Body slender, elytra nealy parallel-sided	*Orthogonius thailandensis* Tian & Deuve, 2006
–	Body stout, elytra distinctly expanded at sides	*Orthogonius brancuccii* Tian & Deuve, 2006

**Figures 26–30.**
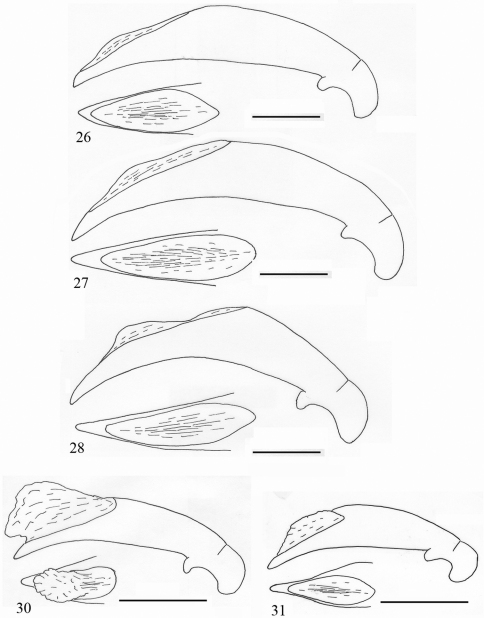
Aedeagus of *Orthogonius* spp. (lateral view, and apex in dorsal view) **26** *Orthogonius thaiensis* Tian & Deuve **27** *Orthogonius pangi* Tian & Deuve **28** *Orthogonius huananoides* Tian & Deuve **29** *Orthogonius nahaeo* Tian & Deuve **30** *Orthogonius loeicus* Tian & Deuve. Scale bar: 1 mm.

**Figures 31–37.**
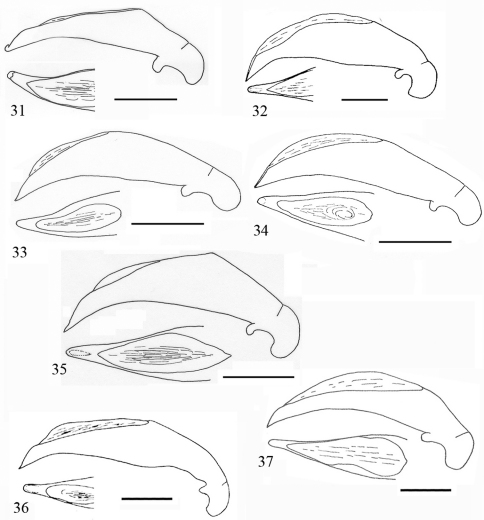
Aedeagus of *Orthogonius* spp. (lateral view, and apex in dorsal view) **31** *Orthogonius loeiensis* Tian & Deuve **32** *Orthogonius kirirom* Tian & Deuve **33** *Orthogonius pachlatkoi* Tian & Deuve **34** *Orthogonius siamensis* Tian & Deuve **35** *Orthogonius chiangensis* Tian & Deuve **36** *Orthogonius pseudolongicornis* Tian & Deuve **37** *Orthogonius longicornis* Chaudoir. Scale bar: 1 mm.

## Discussion and conclusions

Our result shows that Thailand has one of the most diverse *Orthogonius* faunas in the world, comprised of 30 recorded species. This ranks Thailand behind Indonesia with 54 species and India with 46 species, despite the fact the total area of Thailand is much less than that of these two other countries. Malaise traps are mainly used to collect Hymenoptera and Diptera, but they are also efficient when used to catch beetles like *Orthogonius* species that are strong fliers. The majority of the specimens collected in the course of this study were collected in Malaise traps.

**Figure 38.**
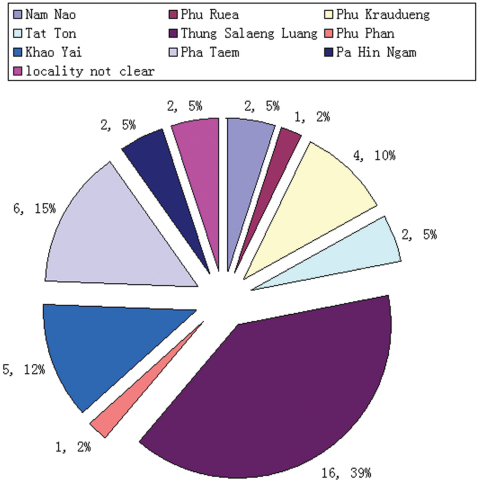
Species number and composition (%) of *Orthogonius* in different national parks in Thailand

*Orthogonius* specimens were only collected in ten of the 25 national parks sampled in Thailand. Only one species was collected in two parks, *viz.* *Orthogonius longicornis* in Phu Phan and *Orthogonius* sp. in Phu Ruea; two species were collected in three parks (Nam Nao, Tat Ton and Pha Hin Ngam); and four, five and six species were collected in Phu Kraudueng, Khao Yai and Pha Taem, respectively. Thung Salaeng Luang National Park holds the richest fauna for *Orthogonius*, with 16 species recorded from that park (Figure 38).

Almost all records of *Orthogonius* species from the TIGER project provide new distribution records for the genus. Detailed collecting data make it possible to analyze the geographical distribution patterns of species in the genus *Orthogonius* in Thailand. Although *Orthogonius* beetles are able to fly, their dispersal ability appears to be limited because the majority of the species of this genus represented in Thailand are endemic, with just a few (six) species known to occur also in other countries nearby, such as Vietnam, Cambodia and China.

## Supplementary Material

XML Treatment for Orthogonius taghavianae

XML Treatment for Orthogonius coomanioides

XML Treatment for Orthogonius similaris

XML Treatment for Orthogonius setosopalpiger

XML Treatment for Orthogonius pangi

XML Treatment for Orthogonius huananoides

XML Treatment for Orthogonius gracililamella

XML Treatment for Orthogonius pseudochaudoiri

XML Treatment for Orthogonius constrictus

XML Treatment for Orthogonius pinophilus

XML Treatment for Orthogonius vari

XML Treatment for Orthogonius kirirom

XML Treatment for Orthogonius leoeinsis

XML Treatment for Orthogonius thailandensis

XML Treatment for Orthogonius pseudolongicornis

XML Treatment for Orthogonius longicornis

XML Treatment for Orthogonius nahaeo

XML Treatment for Orthogonius siamensis

XML Treatment for Orthogonius mouhoti

XML Treatment for Orthogonius variabilis
